# A Relaxed Interior Point Method for Low-Rank Semidefinite Programming Problems with Applications to Matrix Completion

**DOI:** 10.1007/s10915-021-01654-1

**Published:** 2021-10-11

**Authors:** Stefania Bellavia, Jacek Gondzio, Margherita Porcelli

**Affiliations:** 1 Dipartimento di Ingegneria Industriale, Università degli Studi di Firenze, viale Morgagni 40, 50134 Firenze, Italy; 2 School of Mathematics, The University of Edinburgh, James Clerk Maxwell Building, The King’s Buildings, Peter Guthrie Tait Road, Edinburgh, EH9 3FD UK; 3 Dipartimento di Matematica, Università di Bologna, Piazza di Porta San Donato 5, 40126 Bologna, Italy

**Keywords:** Semidefinite programming, Interior point algorithms, Low rank, Matrix completion problems, 90C22, 90C51, 65F10, 65F50

## Abstract

A new relaxed variant of interior point method for low-rank semidefinite programming problems is proposed in this paper. The method is a step outside of the usual interior point framework. In anticipation to converging to a low-rank primal solution, a special nearly low-rank form of all primal iterates is imposed. To accommodate such a (restrictive) structure, the first order optimality conditions have to be relaxed and are therefore approximated by solving an auxiliary least-squares problem. The relaxed interior point framework opens numerous possibilities how primal and dual approximated Newton directions can be computed. In particular, it admits the application of both the first- and the second-order methods in this context. The convergence of the method is established. A prototype implementation is discussed and encouraging preliminary computational results are reported for solving the SDP-reformulation of matrix-completion problems.

## Introduction

We are concerned with an application of an interior point method (IPM) for solving large, sparse and specially structured positive semidefinite programming problems (SDPs).

Let $S{\mathbb {R}}^{n\times n}$ denote the set of real symmetric matrices of order *n* and let $U \bullet V$ denote the inner product between two matrices, defined by $trace (U^T V)$. Consider the standard semidefinite programming (SDP) problem in its primal form

1.1 $$\begin{aligned} \begin{array}{ll} \min &{} C \bullet X \\ \text{ s.t. } &{} A_i \bullet X=b_i\;\;\; i=1,\ldots ,m \\ &{} X \succeq 0, \\ \end{array} \end{aligned}$$

where $A_i, C \in S{\mathbb {R}}^{n\times n}$ and $b\in {\mathbb {R}}^m$ are given and $X \in S{\mathbb {R}}^{n\times n}$ is unknown and assume that matrices $A_i, i=1,2,\dots ,m$ are linearly independent, that is $\sum _{i=1}^m d_i A_i = 0$ implies $d_i=0$, $i=1,\ldots ,m$. The dual form of the SDP problem associated with (1.1) is:

1.2 $$\begin{aligned} \begin{array}{ll} \max &{} b^T y \\ \text{ s.t. } &{} \sum \limits _{i=1}^m y_i A_{i} + S = C \\ &{} S \succeq 0, \end{array} \end{aligned}$$

where $$y \in {\mathbb {R}}^m$$ and $$S \in S{\mathbb {R}}^{n\times n}$$.

The number of applications which involve semidefinite programming problems as a modelling tool is already impressive [40, 44] and is still growing. Applications include problems arising in engineering, finance, optimal control, power flow, various SDP relaxations of combinatorial optimization problems, matrix completion or other applications originating from modern computational statistics and machine learning. Although the progress in the solution algorithms for SDP over the last two decades was certainly impressive (see the books on the subject [2, 15]), the efficient solution of general semidefinite programming problems still remains a computational challenge.

Among various algorithms for solving (linear) SDPs, interior point methods stand out as reliable algorithms which enjoy enviable convergence properties and usually provide accurate solutions within reasonable time. However, when sizes of SDP instances grow, traditional IPMs which require computing exact Newton search directions hit their limits. Indeed, the effort required by the linear algebra in (standard) IPMs may grow as fast as ${{\mathcal {O}}}(n^6)$.

Although there exists a number of alternative approaches to interior point methods, such as for example [8, 9, 30], which can solve certain SDPs very efficiently, they usually come with noticeably weaker convergence guarantees. Therefore there is a need to develop faster IPM-based techniques which could preserve some of the excellent theoretical properties of these methods, but compromise on the other features in quest for practical computational efficiency. Customized IPM methods have been proposed for special classes of problems. They take advantage of sparsity and structure of the problems, see e.g. [4, 5, 21, 31, 36, 41] and the references in [1].

In this paper we focus on problems in which the primal variable *X* is expected to be *low-rank* at optimality. Such situations are common in relaxations of combinatorial optimization problems [5], for example in maximum cut problems [22], as well as in matrix completion problems [11], general trust region problems and quadratically constrained quadratic problems in complex variables [34]. We exploit the structure of the sought solution and relax the rigid structure of IPMs for SDP. In particular we propose to weaken the usual connection between the primal and dual problem formulation and exploit any special features of the primal variable *X*. However, the extra flexibility added to the interior point method comes at a price: the worst-case polynomial complexity has to be sacrificed in this case.

Rank plays an important role in semidefinite programming. For example, every polynomial optimization problem has a natural SDP relaxation, and this relaxation is exact when it possesses a rank-1 solution [34]. On the other hand, for any general problem of the form (1.1), there exists an equivalent formulation where an additional bound *r* on the rank of *X* may be imposed as long as *r* is not too small [9]. More specifically, under suitable assumptions, there exists an optimal solution $X^*$ of (1.1) with rank *r* satisfying $r(r+1)/2 \le m$. There have been successful attempts to identify low rank submatrices in the SDP matrices and eliminate them with the aim to reduce the rank and hence the difficulty of solving an SDP. A technique called *facial reduction* [26] has been analysed and demonstrated to work well in practice. Interestingly, when positive semidefinite programs are solved using interior-point algorithms, then because of the nature of logarithmic barrier function promoting the presence of nonzero eigenvalues, the primal variable *X* typically converges to a maximum-rank solution [24, 34]. However, in this paper we aim at achieving the opposite. We want to design an interior point method which drives the generated sequence of iterates to converge to a low-rank solution. We assume that constraint matrices are sparse and we search for a solution *X* of rank *r* of the form $X= U U^T$ with $U\in {\mathbb {R}}^{n\times r}$.

Special low-rank structure of *X* may be imposed directly in problem (1.1), but this excludes the use of an interior point algorithm (which requires all iterates *X* to be strictly positive definite). Burer and Monteiro [8, 9] and their followers [6, 7] have used such an approach with great success. Namely, they have substituted $U U^T$ for *X* in (1.1) and therefore have replaced it with the following nonlinear programming problem

1.3 $$\begin{aligned} \begin{array}{ll} \min &{} C \bullet (U U^T) \\ \text{ s.t. } &{} A_i\bullet (U U^T) = b_i \;\;\; i=1,\ldots ,m, \end{array} \end{aligned}$$

with $U \in {\mathbb {R}}^{n \times r}$. Although such transformation removes the difficult positive definiteness constraint (it is implicit as $X = U U^T$), the difficulty is shifted elsewhere as both the objective and constraints in (1.3) are no longer linear, but instead quadratic and in general non-convex. In comparison with a standard IPM the method proposed in [8, 9] and applied to solve large-scale problems enjoys substantially reduced memory requirements and very good efficiency and accuracy. However, due to nonconvexity of (1.3), local methods may not always recover the global optimum. In [6, 7] authors showed that, despite the non-convexity, first- and second-order necessary optimality conditions are also sufficient, provided that rank *r* is large enough and constraints satisfy some regularity conditions. That is, when applied to several classes of SDPs, the low-rank Burer–Monteiro formulation is very unlikely to converge to any spurious local optima.

In this paper we propose a different approach. We would like to preserve as many of the advantageous properties of interior point methods as possible and expect to achieve it by (i) working with the original problem (1.1) and (ii) exploiting the low-rank structure of *X*. Knowing that at optimality *X* is low-rank we impose a special form of the primal variable throughout the interior point algorithm

$$\begin{aligned} X = \mu I_n + U U^T, \end{aligned}$$

with $U\in {\mathbb {R}}^{n\times r}$, for a given $r>0$ and $\mu$ denoting the barrier term. Hence *X* is full rank (as required by IPM), but approaches the low-rank matrix as $\mu$ goes to zero. Imposing such special structure of *X* offers an advantage to an interior point algorithm: it can work with an object of size *nr* rather than a full rank *X* of size $n^2$. We have additionally considered an adaptive choice of *r* assuming that this rank may not be known a priori. Indeed, the method can start with *r* equal to 1 or 2 and gradually increase *r* to the necessary minimum rank (*target rank*). Remarkably, the method can also handle problems with nearly-low-rank solution, as the primal variable is not assumed to be low-rank along the iterations, but it is gradually pushed to a low-rank matrix. Finally, the presence of the *perturbation term* $\mu I$ allows to deal with possibly noisy right-hand side *b* as well. We also further relax the rigid IPM structure. Starting from a dual feasible approximation, we dispose of dual slack variable *S* and avoid computations which would involve large Kronecker product matrices of dimension $n^2 \times n^2$ (and that in the worst case might require up to ${{\mathcal {O}}}(n^6)$ arithmetic operations). We investigate the use of both first- and second-order methods for the step computation and devise matrix-free implementations of the linear algebra phase arising in the second-order method. Such implementations are well-suited to the solution of SDP relaxations of matrix completion problems [13].

The paper is organised as follows. After a brief summary of notation used in the paper provided at the end of this section, in Sect. 2 we present the general framework and deliver some theoretical insights into the proposed method. In Sect. 3 we explain the mechanism which allows to adaptively reveal the rank of the minimum rank solution matrix *X*. The proposed approach offers significant flexibility in the way how Newton-like search directions are computed. They originate from a solution of a least squares problem. We see it in detail in Sect. 4. Next, in Sect. 5 we discuss the properties of low-rank SDPs arising in matrix completion problems and in Sect. 6 we present preliminary computational results obtained with a prototype Matlab implementation of the new algorithm. We also provide a comparison of its efficiency against OptSpace [28, 29] when both methods are applied to various instances of matrix completion problems. Finally, in Sect. 7 we give our conclusions. “Appendix A” contains some notes on the Kronecker product of two matrices and on matrix calculus.

**Notation** The norm of the matrix associated with the inner product between two matrices $U \bullet V = trace (U^T V)$ is the Frobenius norm, written $\Vert U\Vert _F := (U\bullet U )^{1/2}$, while $\Vert U\Vert _2$ denotes the L$_2$-operator norm of a matrix. Norms of vectors will always be Euclidean. The symbol $I_p$ denotes the identity matrix of dimension $p\times p$.

Let ${{\mathcal {A}}}$ be the linear operator ${{\mathcal {A}}}: S{\mathbb {R}}^n\rightarrow {\mathbb {R}}^m$ defined by

$$\begin{aligned} {{\mathcal {A}}}(X)=(A_i\bullet X)_{i=1}^m\in {\mathbb {R}}^m, \end{aligned}$$

with $A_i \in S{\mathbb {R}}^{n\times n}$, then its transposition ${{\mathcal {A}}}^T$

$$\begin{aligned} {{\mathcal {A}}}^Tv=\sum _{i=1}^m v_i A_i. \end{aligned}$$

Moreover, let $A^T$ denote the matrix representation of ${{\mathcal {A}}}^T$ with respect to the standard bases of ${\mathbb {R}}^n$, that is

1.4 $$\begin{aligned} A^T := [vec(A_1), vec(A_2), \dots ,vec(A_m)] \in {\mathbb {R}}^{n^2 \times m }, \end{aligned}$$

and

$$\begin{aligned} {{\mathcal {A}}}(X) = A\, vec(X) \quad \text{ and } \quad {{\mathcal {A}}}^Tv=mat(A^{T} v), \end{aligned}$$

where *mat* is the “inverse” operator to *vec* (i.e., $mat(vec(A_i)) = A_i \in S{\mathbb {R}}^{n\times n}$) and the *vec* operator is such that *vec*(*A*) is the vector of columns of *A* stacked one under the other.

## Relaxed Interior Point Method for Low-Rank SDP

Interior point methods for semidefinite programming problems work with the perturbed first-order optimality conditions for problems (1.1)–(1.2) given by:

2.1 $$\begin{aligned} F_\mu (X,y,S) = \left( \begin{array}{c} {{\mathcal {A}}}^T y + S - C \\ {{\mathcal {A}}} (X) - b \\ XS-\mu I_n \end{array} \right) =0, \ \mu > 0, \ S \succeq 0 \ X \succeq 0. \end{aligned}$$

A general IPM involves a triple (*X*, *y*, *S*), performs steps in Newton direction for (2.1), and keeps its subsequent iterates in a neighbourhood of the central path [2, 15]. The convergence is forced by gradually reducing the barrier term $\mu$. However, having in mind the idea of converging to a low-rank solution, we find such a structure rather restrictive and wish to relax it. This is achieved by removing explicit *S* from the optimality conditions and imposing a special structure of *X*.

Substituting $S = C - {{\mathcal {A}}}^Ty$ from the first equation into the third one, we get

2.2 $$\begin{aligned} \left( \begin{array}{c} {{\mathcal {A}}} (X) - b \\ X (C - {{\mathcal {A}}}^T y) -\mu I_n \end{array} \right) =0, \ \mu > 0, \ C - {{\mathcal {A}}}^Ty \succeq 0,\ X \succeq 0. \end{aligned}$$

Next, following the expectation that at optimality *X* has rank *r*, we impose on *X* the following special structure

2.3 $$\begin{aligned} X = \mu I_n + U U^T, \end{aligned}$$

with $U \in {\mathbb {R}}^{n \times r}$, for a given $r>0$. We do not have any guarantee that there exists a solution of (2.2) with such a structure, but we can consider the least-square problem:

2.4 $$\begin{aligned} \min _{U,y} \phi _\mu (U,y) := \frac{1}{2}\Vert F^r_{\mu }(U,y)\Vert ^2, \end{aligned}$$

where $F^r_{\mu }(U,y): {\mathbb {R}}^{n\times r } \times {\mathbb {R}}^{m}\rightarrow {\mathbb {R}}^{n^2+m}$ is given by

2.5 $$\begin{aligned} F^r_{\mu }(U,y)= \left( \begin{array}{c} {{\mathcal {A}}} (\mu I_n + U U^T) - b \\ vec((\mu I_n + U U^T) (C - {{\mathcal {A}}}^T y) - \mu I_n ) \end{array} \right) , \ \mu > 0. \end{aligned}$$

The nonlinear function $F^r_{\mu }(U,y)$ has been obtained substituting $X = \mu I_n + U U^T$ in (2.2) after vectorization of the second block. The associated system $F^r_{\mu }(U,y)=0$ is overdetermined with $(m+n^2)$ equations and ($nr+m$) unknowns (*U*, *y*). In the following, for the sake of simplicity, we identify ${\mathbb {R}}^{n\times r} \times {\mathbb {R}}^{m}$ with ${\mathbb {R}}^{nr+m}$.

It is worth mentioning at this point that the use of least-squares type solutions to an overdetermined systems arising in interior point methods for SDP was considered in [16, 32]. Its primary objective was to avoid symmetrization when computing search directions and the least-squares approach was applied to a standard, complete set of perturbed optimality conditions (2.1).

We propose to apply to problem (2.4) a similar framework to that of interior point methods, namely: Fix $\mu$, iterate on a tuple (*U*, *y*), and make steps towards a solution to (2.4). This opens numerous possibilities. One could for example compute the search directions for both variables at the same time, or alternate between the steps in *U* and in *y*.

Bearing in mind that (2.1) are the optimality conditions for (1.1) and assuming that a rank *r* optimal solution of (1.1) exists, we will derive an upper bound on the optimal residual of the least-squares problem (2.4). Assume that a solution $(X^*,y^*,S^*)$ of the KKT conditions exists such that $X^*=U^*(U^*)^T$, $U^* \in {\mathbb {R}}^{n \times r}$, that is

2.6 $$\begin{aligned} \begin{array}{l} {{\mathcal {A}}} (U^*(U^*)^T) = b \\ S^* = C - {{\mathcal {A}}}^T y^* \succeq 0 \\ U^*(U^*)^T S^* = 0. \end{array} \end{aligned}$$

Then evaluating (2.5) at $(U^*,y^*)$ and using (2.6) we get

$$\begin{aligned} F^r_{\mu } (U^*,y^*) = \left( \begin{array}{c} {{\mathcal {A}}} (\mu I_n)+ {{\mathcal {A}}}(U^* (U^*)^T) - b\\ vec( (\mu I_n + U^* (U^*)^T) (C - {{\mathcal {A}}}^T y^*) -\mu I_n ) \end{array} \right) = \left( \begin{array}{c} \mu {{\mathcal {A}}} ( I_n) \\ \mu \, vec(S^* - I_n) \end{array} \right) . \end{aligned}$$

Consequently, we obtain the following upper bound for the residual of the least-squares problem (2.4):

2.7 $$\begin{aligned} \phi _{\mu }(U^*,y^*)= & {} \frac{1}{2} \Vert {{\mathcal {A}}} (U^* (U^*)^T+\mu I_n) - b\Vert ^2 + \frac{1}{2}\Vert (U^*(U^*)^T+\mu I_n) (C - {{\mathcal {A}}}^T y^*) - \mu I_n\Vert _F^2 \nonumber \\= & {} \omega ^* \mu ^2, \end{aligned}$$

where

2.8 $$\begin{aligned} \omega ^*=\frac{1}{2}\Vert {{\mathcal {A}}} (I_n)\Vert _2^2 + \frac{1}{2}\Vert S^*-I_n\Vert ^2_F. \end{aligned}$$

Assuming to have an estimate of $\omega ^*$ we are now ready to sketch in Algorithm 1 the general framework of a new *relaxed* interior point method.

To start the procedure we need an initial guess $(U_0,y_0)$ such that $U_0$ is full column rank and $S_0 = C - {{\mathcal {A}}}^T y_0$ is positive definite, and an initial barrier parameter $\mu _0 > 0$. At a generic iteration *k*, given the current barrier parameter $\mu _k>0$, we compute an approximate solution $(U_k,{\bar{y}}_k)$ of (2.4) such that $\phi _{\mu _k}(U_k,{\bar{y}}_k)$ is below $\mu _k^2 \omega ^*$. Then, the dual variable $y_k$ and the dual slack variable $S_k$ are updated as follows:

$$\begin{aligned} \begin{array}{l} y_k = y_{k-1} + \alpha _k ({\bar{y}}_k - y_{k-1}) \\ S_k = C - {{\mathcal {A}}}^T y_k = S_{k-1}-\alpha _k {{\mathcal {A}}}^T ({\bar{y}}_k - y_{k-1}) \\ \end{array} \end{aligned}$$

with $\alpha _k \in (0,1]$ such that $S_k$ remains positive definite. We draw the reader’s attention to the fact that although the dual variable *S* does not explicitly appear in optimality conditions (2.2) or (2.5), we do maintain it as the algorithm progresses and make sure that $(S_k,y_k)$ remains dual feasible. Finally, to complete the major step of the algorithm, the barrier parameter is reduced and a new iteration is performed.

Note that so far we have assumed that there exists a solution to (1.1) of rank *r*. In case such a solution does not exist the optimal residual of the least-squares problem is not guaranteed to decrease as fast as $\mu _k^2$. This apparently adverse case can be exploited to design an adaptive procedure that increases/decreases *r* without requiring the knowledge of the solution’s rank. This approach will be described in Sect. 3.
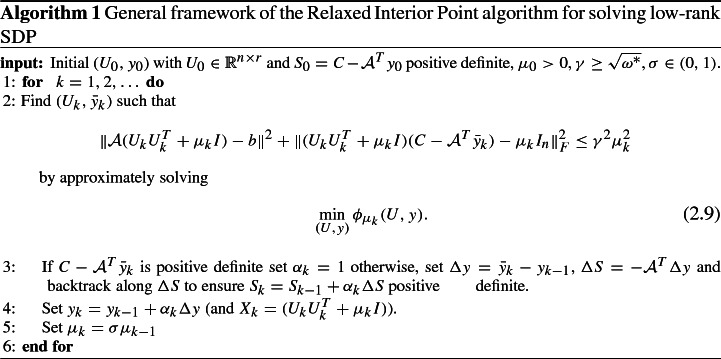


In the remaining part of this section we state some of the properties of the Algorithm which are essential to make it work in practice.

First we note that dual constraint is always satisfied by construction and the backtracking process at Line 3 is well-defined. This is proved in Lemma 4 of [4] which is repeated below for sake of reader’s convenience.

### Lemma 2.1

Let $\Delta S$ be computed in Step 3 of Algorithm 1 at iteration *k* and $S_{k-1}$ be computed at the previous iteration $k-1$. Then, there exists $\alpha _k\in (0, 1]$ such that $S_k=S_{k-1}+\alpha _k \Delta S$ is positive definite.

### Proof

Assume that $C - {{\mathcal {A}}}^T {\bar{y}}_k$ is not positive definite, otherwise $\alpha _k=1$. Noting that $S_{k-1} \succ 0$ by construction, it follows that $\Delta S$ is indefinite and $S_{k-1} + \alpha _k \Delta S \succ 0$ whenever $\alpha _k$ is sufficiently small. In particular, since $S_{k-1} + \alpha _k \Delta S = S_{k-1}^{1/2} (I_n + \alpha _k S_{k-1}^{-1/2} \Delta S S_{k-1}^{-1/2}) S_{k-1}^{1/2}$, the desired result holds with

$$\begin{aligned} \alpha _k< \frac{-1}{\lambda _{\min }(S_{k-1}^{-1/2} \Delta S S_{k-1}^{-1/2})}. \end{aligned}$$

$$\square $$

Note that if backtracking is needed (i.e. $\alpha _k<1$) to maintain the positive definiteness of the dual variable, then after updating $S_k$ in Step 5 the centrality measure $\Vert X_k S_k - \mu _k I_n\Vert$ may increase and it is not guaranteed to remain below $\gamma \mu _k$. Indeed, by setting $S_k = {\bar{S}}_k - (1-\alpha _k) \Delta S$ with ${\bar{S}}_k = C - {{\mathcal {A}}}^T {\bar{y}}_k$, we have:

2.10 $$\begin{aligned} \Vert X_k S_k - \mu _k I_n\Vert ^2_F \le \gamma ^2 \mu ^2_k + (1-\alpha _k)^2 \Vert X_k\Delta S\Vert ^2_F - 2 (1-\alpha _k) (X_k {\bar{S}}_k - \mu _k I_n)\bullet (X_k \Delta S),\nonumber \\ \end{aligned}$$

that is the centrality measure may actually increase along the iterations whenever $\alpha _k$ does not approach one as $\mu _k$ goes to zero. In the following we analyse the convergence properties of Algorithm 1 when this adverse situation does not occur, namely under the following assumption:

### Assumption 1

Assume that there exists ${\bar{k}}>0$ such that $\alpha _k= 1$ for $k\ge {\bar{k}}$.

To the best of authors knowledge, it does not seem possible to demonstrate that eventually $\alpha _k$ is equal to one. This is because we impose a special form of *X* in (2.3) and make only a weak requirement regarding the proximity of the iterate to the central path:

2.11 $$\begin{aligned} \Vert (U_kU_k^T+\mu _k I) (C - {{\mathcal {A}}}^T {\bar{y}}_k) - \mu _k I_n\Vert _F \le \gamma \mu _k \end{aligned}$$

with $\gamma$ possibly greater than one.

### Proposition 2.2

Let Assumption 1 hold. Assume that a solution of rank *r* of problem (1.1) exists and that the sequence $\{U_k,y_k\}$ admits a limit point $(U^\dagger , y^\dagger )$. Then,

- $X^\dagger =U^\dagger (U^\dagger )^T$ is primal feasible,
- $$X^\dagger S^\dagger =0$$ with $$ S^\dagger = C- {{\mathcal {A}}}^T y^\dagger $$,
- $S^\dagger$ is positive semidefinite.

### Proof

Assume for the sake of simplicity that the whole sequence is converging to $(U^\dagger , y^\dagger )$. Taking into account that $\lim _{k\rightarrow \infty } \mu _k=0$, it follows $\lim _{k\rightarrow \infty } U_k (U_k)^T + \mu _k I = (U^\dagger )(U^\dagger )^T$. Then $X^\dagger = (U^\dagger )(U^\dagger )^T$ has at most rank *r* and it is feasible as

$$\begin{aligned} \lim _{k\rightarrow \infty }\Vert {{\mathcal {A}}} (U_k U_k ^T+\mu _k I) - b\Vert \le \lim _{k\rightarrow \infty }\gamma \mu _k=0. \end{aligned}$$

Moreover, from (2.10) and Assumption 1 it follows

$$\begin{aligned} \lim _{k\rightarrow \infty }\Vert (U_kU_k^T+\mu _k I) (C - {{\mathcal {A}}}^T y_k) -\mu _k I_n\Vert _F = 0, \end{aligned}$$

which implies $X^\dagger S^\dagger =0$ and by construction ensures that $S^\dagger$ is positive semidefinite being a limit point of a sequence of positive definite matrices. $\square$

From the previous proposition it follows that $(X^\dagger , y^{\dagger },S^\dagger )$ solves (2.1). Moreover, $X^\dagger$ has rank *r*, unless $U^\dagger$ is not full column rank. This situation can happen only in the case (1.1) admits a solution of rank smaller than *r*. In what follows for sake of simplicity we assume that the limit point $U^\dagger$ is full column rank.

### Remark

It is worth observing that due to the imposed structure of matrices (2.3) all iterates $X_k$ are full rank, but asymptotically they approach rank *r* matrix. Moreover, the minimum distance of $X_k$ to a rank *r* matrix is given by $\mu _k$, i.e.,

2.12 $$\begin{aligned} \min _{rank(Y)=r} \Vert X_k-Y\Vert _2=\mu _k, \end{aligned}$$

and the primal infeasibility is bounded by $\gamma \mu _k$. This allows us to use the proposed methodology also when the sought solution is close to a rank *r* matrix (“nearly low-rank”) and/or some entries in vector *b* are corrupted with a small amount of noise.

## Rank Updating/Downdating

The analysis carried out in the previous section requires the knowledge of $\gamma \ge \sqrt{\omega ^*}$ and of the rank *r* of the sought solution. As the scalar $\gamma$ is generally not known, at a generic iteration *k* the optimization method used to compute an approximate minimizer of (2.4) is stopped when a chosen first-order criticality measure $\psi _{\mu }$ goes below the threshold $\eta _2 \mu _k$ where $\eta _2$ is a strictly positive constant. This way, the accuracy in the solution of (2.4) increases as $\mu _k$ decreases. For $\psi _{\mu }$, we have chosen $\psi _{\mu }(U,y)=\Vert \nabla \phi _{\mu }(U,y)\Vert _2$.

Regarding the choice of the rank *r*, there are situations where the rank of the sought solution is not known. Below we describe a modification of Algorithm 1 where, starting from a small rank *r*, the procedure adaptively increases/decreases it. This modification is based on the observation that if a solution of rank *r* exists the iterative procedure used in Step 2, should provide a sequence $\{U_k\}$ such that the primal infeasibility also decreases with $\mu _k$. Then, at each iteration the ratio

3.1 $$\begin{aligned} \rho _k = \frac{\Vert {{\mathcal {A}}} (U_k U_k ^T+\mu _k I) - b\Vert _2}{\Vert {{\mathcal {A}}} (U_{k-1} U_{k-1} ^T+\mu _{k-1} I) - b\Vert _2}, \end{aligned}$$

is checked. If this ratio is larger than $\eta _1$, where $\eta _1$ is a given constant in $(\sigma ,1)$ and $\sigma$ is the constant used to reduce $\mu _k$, then the rank *r* is increased by some fixed $\delta _r>0$ as the procedure has not been able to provide the expected decrease in the primal infeasibility. After an update of rank, the parameter $\mu _k$ is not changed and $\delta _r$ extra columns are appended to the current $U_k$. As a safeguard, also a downdating strategy can be implemented. In fact, if after an increase of rank, we still have $\rho _k>\eta _1$ then we come back to the previous rank and inhibit rank updates in all subsequent iterations.

This is detailed in Algorithm 2 where we borrowed the Matlab notation. Variable update_r is an indicator specifying if at the previous iteration the rank was increased (update_r = up), decreased (update_r = down) or left unchanged (update_r = unch).
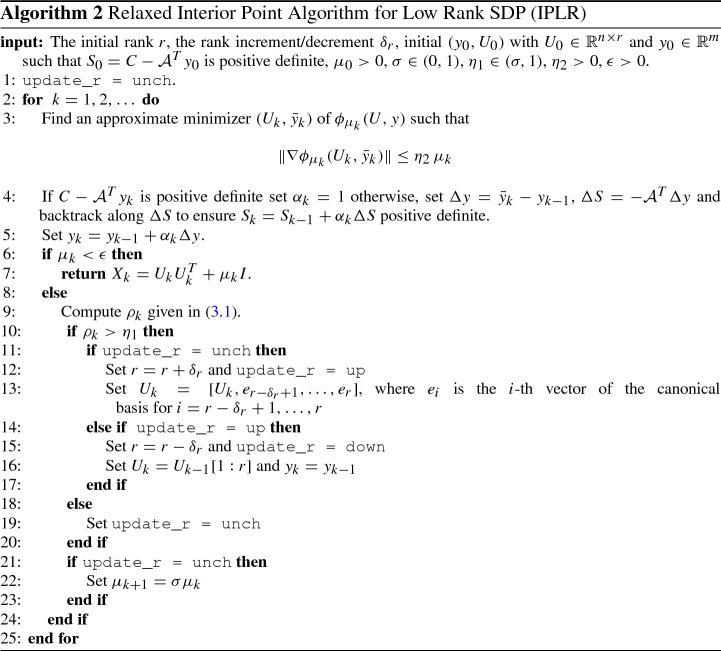


The initial rank *r* should be chosen as the rank of the solution (if known) or as a small value (say 2 or 3) if it is unknown. The dimension of the initial variable $U_0$ is then defined accordingly. Since, for given $\epsilon$ and $\sigma$, the number of iterations to satisfy $\mu _k < \epsilon$ at Line 6 is predefined, the number of rank updates is predefined as well. Therefore, if an estimate of the solution rank is known, one should use it in order to define a suitable initial *r*.

## Solving the Nonlinear Least-Squares Problem

In this section we investigate the numerical solution of the nonlinear least-squares problem (2.4).

Following the derivation rules recalled in “Appendix A”, we compute the Jacobian matrix $J_{\mu _k} \in {\mathbb {R}}^{(n^2+m) \times (nr+m)}$ of $F^r_{\mu _k}$ which takes the following form:

$$\begin{aligned} J_{\mu _k}(U,y) = \left( \begin{array}{cc} A Q &{} 0 \\ ((C - {{\mathcal {A}} }^Ty) \otimes I_n) Q \quad &{} -(I_n \otimes (\mu _k I_n + U U^T)) A^T \end{array} \right) , \end{aligned}$$

where

4.1 $$\begin{aligned} Q = (U\otimes I_n) + (I_n \otimes U) \Pi _{nr} \in {\mathbb {R}}^{n^2\times nr}, \end{aligned}$$

and $\Pi _{nr} \in {\mathbb {R}}^{nr\times nr}$ is the unique permutation matrix such that $vec(B^T) = \Pi _{nr} vec(B)$ for any $B \in {\mathbb {R}}^{n \times r}$, see “Appendix A”.

In order to apply an iterative method for approximately solving (2.4) we need to perform the action of $J_{\mu _k}^T$ on a vector to compute the gradient of $\phi _{\mu _k}$. The action of $J_{\mu _k}$ on a vector is also required in case one wants to apply a Gauss–Newton approach (see Sect. 4.3). In the next section we will discuss how these computations are carried out.

### Matrix–Vector Products with Blocks of $J_{\mu _k}$

First, let us denote the Jacobian matrix blocks as follows:

4.2 $$\begin{aligned} J_{11}= & {} A Q = A (( U \otimes I_n) + (I_n \otimes U) \Pi _{nr}) \in {\mathbb {R}}^{m \times nr} \end{aligned}$$

4.3 $$\begin{aligned} J_{21}= & {} ((C - {{\mathcal {A}} }^Ty) \otimes I_n) Q = (S \otimes I_n) Q \in {\mathbb {R}}^{n^2 \times nr} \end{aligned}$$

4.4 $$\begin{aligned} J_{22}= & {} - (I_n \otimes (\mu _k I_n + U U^T)) A^T = - (I_n \otimes X) A^T \in {\mathbb {R}}^{n^2 \times m}. \end{aligned}$$

Below we will show that despite $J_{\mu _k}$ blocks contain matrices of dimension $n^2\times n^2$, matrix-vector products can be carried out without involving such matrices and the sparsity of the constraint matrices can be exploited. We will make use of the properties of the Kronecker product (A.1)–(A.3) and assume that if $v \in {\mathbb {R}}^{nr}$ and ${\tilde{z}} \in {\mathbb {R}}^{n^2}$ then $mat(v) \in {\mathbb {R}}^{n\times r}$ and $mat({\tilde{z}}) \in {\mathbb {R}}^{n\times n}$.

- Let $v\in {\mathbb {R}}^{nr}$ and $w\in {\mathbb {R}}^m$ and let us consider the action of $J_{11}$ and $J_{11}^T$ on *v* and *w*, respectively:
  4.5 $$\begin{aligned} J_{11} v= & {} {{\mathcal {A}}} ( mat(v) U^T + U mat(v)^T ) = \left( A_i \bullet V \right) _{i=1}^m \end{aligned}$$
  where
  4.6 $$\begin{aligned} V = mat(v) U^T + U mat(v)^T \in {\mathbb {R}}^{n\times n}, \end{aligned}$$
  and
  4.7 $$\begin{aligned} J_{11}^T w= & {} Q^T A^T w= (( U^T \otimes I_n) + \Pi _{nr}^T(I_n \otimes U^T)) A^T w \nonumber \\= & {} (( U^T \otimes I_n) + \Pi _{nr}^T(I_n \otimes U^T)) vec ({{\mathcal {A}}}^T w) \nonumber \\= & {} vec(({{\mathcal {A}}}^T w)U + ({{\mathcal {A}}}^T w)^T U) \nonumber \\= & {} 2 vec(({{\mathcal {A}}}^T w)U) = 2 vec\left( \sum _{i=1}^m w_i A_i U\right) . \end{aligned}$$
- Let $v \in {\mathbb {R}}^{nr}$ and ${\tilde{z}} \in {\mathbb {R}}^{n^2}$ and let us consider the action of $J_{21}^TJ_{21}$ and $J_{21}^T$ on *v* and ${\tilde{z}}$, respectively:
  4.8 $$\begin{aligned} J_{21}^T J_{21} v= & {} Q^T (S^2 \otimes I_n) Q v \nonumber \\= & {} vec( (mat( v) U^T + U mat( v)^T ) S^2 U \nonumber \\&+ S^2 (mat( v) U^T + U mat(v)^T)^T U ) \end{aligned}$$
  and
  4.9 $$\begin{aligned} J_{21}^T {\tilde{z}}= & {} Q^T vec (mat({\tilde{z}})S) = vec(mat({\tilde{z}}) S U + S mat({\tilde{z}})^T U). \end{aligned}$$
- Let $w \in {\mathbb {R}}^{m}$ and ${\tilde{z}} \in {\mathbb {R}}^{n^2}$ and let us consider the action of $J_{22}$ and $J_{22}^T$ on *w* and ${\tilde{z}}$, respectively:
  4.10 $$\begin{aligned} J_{22} w=-(I \otimes X) A^T w= -vec(X {{\mathcal {A}} }^T w) =- vec(X \sum _{i=1}^m w_i A_i) \end{aligned}$$
  and
  4.11 $$\begin{aligned} J_{22}^T{\tilde{z}}=-A(I\otimes X)z=-A vec(X mat({\tilde{z}}))=-{{\mathcal {A}}}(X mat({\tilde{z}}))=-\left( A_i \bullet {\tilde{Z}}\right) _{i=1}^m, \end{aligned}$$
  with
  4.12 $$\begin{aligned} {\tilde{Z}} = (\mu I_n + U U^T)mat({\tilde{z}}). \end{aligned}$$

### Computational Effort Per Iteration

The previous analysis shows that we can perform all products involving Jacobian’s blocks handling only $n\times n$ matrices. Moreover, if matrices $A_i$ are indeed very sparse, their structure can be exploited in the matrix-products in (4.7) and (4.10). (Sparsity has been exploited of course in various implementations of IPM for SDP, see e.g. [20].) Additionally, only few elements of matrices *V* in (4.6) and ${\tilde{Z}}$ in (4.12) need to be involved when products (4.5) and (4.11) are computed, respectively. More precisely, denoting with *nnz*(*A*) the number of nonzero entries of *A*, we need to compute *nnz*(*A*) entries of *V* and ${\tilde{Z}}$ defined in (4.6) and (4.12), respectively. Noting that $mat(v) \in {\mathbb {R}}^{n\times r}$ and $U^T\in {\mathbb {R}}^{r\times n}$, the computation of the needed entries of *V* amounts to (*O*(*nnz*(*A*)*r*) flops. Regarding ${\tilde{Z}}$, the computation of the intermediate matrix ${\hat{W}}= U^T mat({\tilde{z}}) \in {\mathbb {R}}^{r \times n}$ costs $O(n^2r)$ flops and *nnz*(*A*) entries of $U {\hat{W}}$ requires *O*(*nnz*(*A*)*r*) flops.

In Table 1 we provide the estimate flop counts for computing various matrix-vector products with the blocks of Jacobian matrix. We consider the products that are relevant in the computation of the gradient of $\phi _{\mu _k}$ and in handling the linear-algebra phase of the second order method which we will introduce in the next section. From the table, it is evident that the computation of the gradient of $\phi _{\mu _k}$ requires $O(\max \{ nnz(A),n^2\}r+m)$ flops.

**Table 1:** Jacobian’s block times a vector: number of flops

Operation	Cost
$J_{11} v$	$O(nnz(A)( r+1))$
$J_{11}^T w$	*O*(*nnz*(*A*)*r*)
$J_{21}^T J_{21} v$	$O(n^2 r)$
$J_{21}^T {\tilde{z}}$	$O(n^2 r)$
$J_{22} w$	*O*(*n*(*nnz*(*A*)))
$J_{22}^T {\tilde{z}}$	$O(n^2+nnz(A))r$

Below we provide an estimate of a computational effort required by the proposed algorithm under mild assumptions:

1. $$nnz(A)=O(n^2)$$,
2. at Step 3 of Algorithm 2 a line-search first-order method is used to compute an approximate minimizer $(U_k, {\bar{y}}_k)$ of $\phi _{\mu _k}(U,y)$ such that
  $$\begin{aligned} \Vert \nabla \phi _{\mu _k}(U_k,{\bar{y}}_k)\Vert \le \eta _2\, \mu _k. \end{aligned}$$

Taking into account that a line-search first-order method requires in the worst-case $O(\mu _k^{-2})$ iterations to achieve $\Vert \nabla \phi (U_k,y_k)\Vert \le \mu _k$ [23], the computational effort of iteration *k* of Algorithm 2 is $O(\mu _k^{-2}(n^2 r+m))$ in the worst-case. Therefore, when *n* is large, in the early/intermediate stage of the iterative process, this effort is significantly smaller than $O(n^6)$ required by a general purpose interior-point solver [2, 15] or $O(n^4)$ needed by the specialized approach for nuclear norm minimization [36]. We stress that this is a worst-case analysis and in practice we expect to perform less than $O(\mu _k^{-2})$ iterations of the first-order method. In case the number of iterations is of the order of *O*(*n*) the computational effort per iteration of Algorithm 2 drops to $O(n^3 r+nm)$.

Apart from all operations listed above the backtracking along $\Delta S$ needs to ensure that $S_k$ is positive definite (Algorithm 1, Step 4) and this is verified by computing the Cholesky factorization of the matrix $S_{k-1}+\alpha _k \Delta S$, for each trial steplength $\alpha _k$. If the dual matrix is sparse, i.e. when matrices $A_i$, $i=1,\ldots ,m$ and *C* share the sparsity patterns [43], a sparse Cholesky factor is expected. Note that the structure of dual matrix does not change during the iterations, hence reordering of $S_0$ can be carried out once at the very start of Algorithm 2 and then may be reused to compute the Cholesky factorization of $S_{k-1}+\alpha _k \Delta S$ at each iteration.

### Nonlinear Gauss–Seidel Approach

The crucial step of our interior point framework is the computation of an approximate solution of the nonlinear least-squares problem (2.4). To accomplish the goal, a first-order approach as well as a Gauss–Newton method can be used. However, in this latter case the linear algebra phase becomes an issue, due to the large dimension of the Jacobian. Here, we propose a Nonlinear Gauss–Seidel method. We also focus on the linear algebra phase and present a matrix-free implementation well suited for structured constraint matrices as those arising in the SDP reformulation of matrix completion problems [13]. The adopted Nonlinear Gauss–Seidel method to compute $(U_k,{\bar{y}}_k)$ at Step 3 of Algorithm 2 is detailed in Algorithm 3.
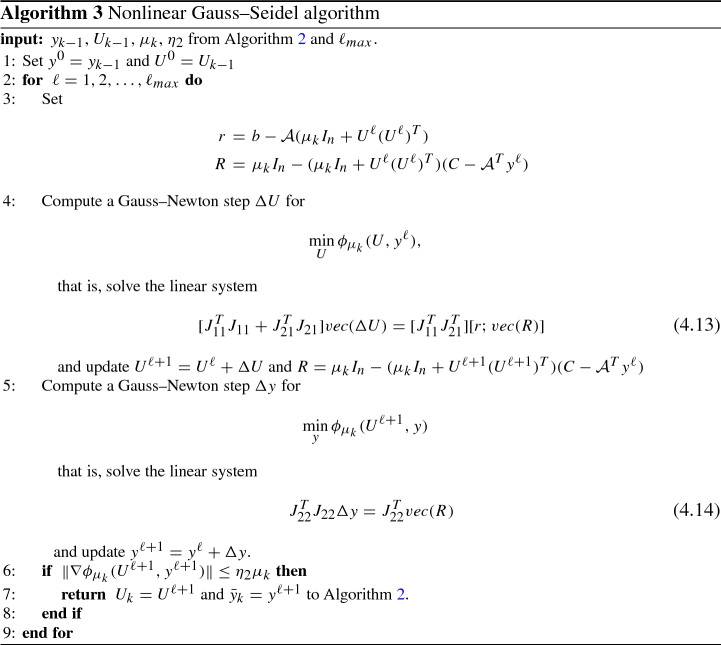
 The computational bottleneck of the procedure given in Algorithm 3 is the solution of the linear systems (4.13) and (4.14). Due to their large dimensions we use a CG-like approach. The coefficient matrix in (4.13) takes the form:

$$\begin{aligned} J_{11}^TJ_{11}\!+\! J_{21}^TJ_{21}\!=\!Q_k^TA^TA Q_k \!+\! Q_k^T (S_k^2 \otimes I_n) Q_k \!=\! Q_k^T (A^TA \!+\! (S_k^2 \otimes I_n)) Q_k \in {\mathbb {R}}^{nr \times nr}, \end{aligned}$$

and it is positive semidefinite as $Q_k$ may be rank deficient. We can apply CG to (4.13) which is known to converge to the minimum norm solution if starting from the null approximation [25]. Letting ${\bar{v}} \in {\mathbb {R}}^{nr}$ be the unitary eigenvector associated to the maximum eigenvalue of $Q_k^T (A^TA + (S_k^2 \otimes I_n)) Q_k$ and ${\bar{w}}=Q_k {\bar{v}}$ we have:

$$\begin{aligned} \lambda _{max}(Q_k^T (A^TA + (S_k^2 \otimes I_n)) Q_k)= & {} {\bar{w}}^T (A^TA + (S_k^2 \otimes I_n))) {\bar{w}}\\\le & {} \lambda _{max} (A^TA + (S_k^2 \otimes I_n))) \Vert {\bar{w}}\Vert ^2\\\le & {} ((\sigma _{max}(A))^2+(\lambda _{max}(S_k))^2)(\sigma _{max}(Q_k))^2. \end{aligned}$$

**Fig. 1:**
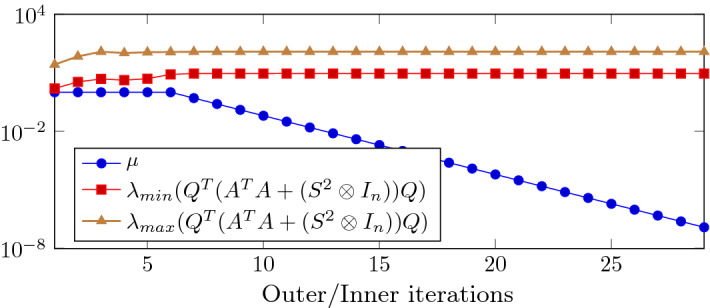
The minimum nonzero eigenvalue and the maximum eigenvalue of the coefficient matrix of (4.13) and $\mu _k$ (semilog scale) versus Outer/Inner IPLR-GS iterations. Data: ${\hat{n}}=100$, $r=4$

Moreover, using 4.1 we derive the following bound

$$\begin{aligned} \sigma _{max}(Q_k)= & {} \sigma _{max}((U_k\otimes I_n) + (I_n \otimes U_k) \Pi _{nr})\\\le & {} \sigma _{max}(U\otimes I_n) + \sigma _{max} ((I_n \otimes U) \Pi _{nr})\\\le & {} 2 \sigma _{max}(U), \end{aligned}$$

as $\sigma _{max}(U\otimes I_n) =\sigma _{max}(U)$ and $\sigma _{max}((U\otimes I_n) )\Pi _{nr})\le \sigma _{max}(U)$. Since both the maximum eigenvalue of $S_k$ and the maximum singular value of $U_k$ are expected to stay bounded from above, we conclude that the maximum eigenvalue of $J_{11}^TJ_{11}+ J_{21}^TJ_{21}$ remains bounded. The smallest nonzero eigenvalue may go to zero at the same speed as $\mu _k^2$. However, in case of SDP reformulation of matrix completion problems, the term $A^TA$ acts as a regularization term and the smallest nonzero eigenvalue of $J_{11}^TJ_{11}+ J_{21}^TJ_{21}$ remains bounded away from $\mu _k$ also in the later iterations of the interior point algorithm. We will report on this later on, in the numerical results section (see Fig. 1).

Let us now consider system (4.14). The coefficient matrix takes the form

4.15 $$\begin{aligned} J_{22}^T J_{22} = A (I_n \otimes X_k^2) A^T \in {\mathbb {R}}^{m\times m}, \end{aligned}$$

and it is positive definite. We repeat the reasoning applied earlier to $J_{11}^TJ_{11}+ J_{21}^TJ_{21}$ and conclude that

$$\begin{aligned} \lambda _{max}(J_{22}^T J_{22})\le (\lambda _{max}(X_k))^2(\sigma _{max}(A))^2. \end{aligned}$$

Analogously we have

$$\begin{aligned} \lambda _{min}(J_{22}^T J_{22})\ge (\lambda _{min}(X_k))^2(\sigma _{min}(A))^2. \end{aligned}$$

Taking into account that *r* eigenvalues of $X_k$ do not depend on $\mu _k$ while the remaining are equal to $\mu _k$, we conclude that the condition number of $J_{22}^T J_{22}$ increases as ${{\mathcal {O}}}(1/\mu _k^2)$. In the next subsection we will show how this matrix can be preconditioned.

### Preconditioning $J_{22}^TJ_{22}$

In this subsection we assume that matrix $AA^T$ is sparse and easy to invert. At this regard we underline that in SDP reformulation of matrix-completion problems matrices $A_i$ have a very special structure that yields $AA^T=\frac{1}{2}I_m$.

Note that substituting $X_k = \mu _k I_n + U_k U_k^T$ in (4.15) we get

4.16 $$\begin{aligned} A (I_n \otimes X_k^2) A^T= & {} A ( \mu _k^2 I_{n^2} + 2 \mu _k I_n \otimes U_kU_k^T + I_n \otimes (U_kU_k^T)^2 ) A^T \end{aligned}$$

4.17 $$\begin{aligned}= & {} \mu _k^2 AA^T + 2 \mu _k A (I_n \otimes U_kU_k^T) A^T + A(I_n \otimes (U_kU_k^T)^2) A^T \end{aligned}$$

4.18 $$\begin{aligned}= & {} \mu _k^2 AA^T + 2 \mu _k A (I_n \otimes U_kU_k^T) A^T \nonumber \\&+ A (I_n \otimes (U_kU_k^T))(I_n \otimes (U_kU_k^T)) A^T.\end{aligned}$$

Let us consider a preconditioner $P_k$ of the form

4.19 $$\begin{aligned} P_k = \mu _k {AA^T } + Z_k Z_k^T, \end{aligned}$$

with

4.20 $$\begin{aligned} Z_k = A(I_n \otimes (U_kU_k^T)) \in {\mathbb {R}}^{m \times n^2}. \end{aligned}$$

This choice is motivated by the fact that we discard the term $I_n \otimes U_kU_k^T$ from the term $2 \mu _k A (I_n \otimes U_kU_k^T) A^T$ in the expression of $J_{22}^T J_{22}$. In fact, we use the approximation

$$\begin{aligned} \mu _k^2 AA^T + 2 \mu _k A (I_n \otimes U_kU_k^T) A^T\approx \mu _k AA^T. \end{aligned}$$

A similar idea is used in [46]. An alternative choice involves matrix $Z_k$ of a smaller dimension

4.21 $$\begin{aligned} Z_k = A(I_n \otimes U_k) \in {\mathbb {R}}^{m \times nr}. \end{aligned}$$

This corresponds to introducing a further approximation

$$\begin{aligned} A(I_n \otimes (U_kU_k^T))(I_n \otimes (U_kU_k^T)) A^T\approx A(I_n \otimes (U_kU_k^T)) A^T. \end{aligned}$$

We will analyze spectral properties of the matrix $J_{22}^T J_{22}$ preconditioned with $P_k$ defined in (4.19) with $Z_k$ given in (4.20).

#### Theorem 4.1

Let $P_k$ be given in (4.19) with $Z_k$ given in (4.20) and $\sigma _{min}(A)$ and $\sigma _{max}(A)$ denote the minimum and maximum singular values of *A*, respectively. The eigenvalues of the preconditioned matrix $P_k^{-1/2}(A (I \otimes X_k^2) A^T)P_k^{-1/2}$ belong to the interval $(1+\xi _1,1+\xi _2)$ where $\xi _1$ and $\xi _2$ have the following forms:

$$\begin{aligned} \xi _1=\frac{\mu _k(\mu _k-1)(\sigma _{\min }(A))^2}{(\sigma _{\max }(A))^2(\mu _k+(\lambda _{\max }(U_kU_k^T))^2)} \end{aligned}$$

and

$$\begin{aligned} \xi _2=\frac{(\sigma _{max}(A))^2(\mu _k+\lambda _{\max } (U_kU_k^T))}{(\sigma _{\min }(A))^2}. \end{aligned}$$

#### Proof

Note that

$$\begin{aligned} A (I \otimes X_k^2) A^T = \mu _k(\mu _k-1) AA^T + 2 \mu _k A (I_n \otimes U_kU_k^T) A^T + P_k. \end{aligned}$$

Then,

$$\begin{aligned} P_k^{-1/2}(A (I \otimes X_k^2) A^T)P_k^{-1/2} = I + \mu _k P_k^{-1/2}((\mu _k-1)AA^T+2 A (I_n \otimes U_kU_k^T) A^T) P_k^{-1/2}. \end{aligned}$$

Let us denote with $\lambda _M$ and $\lambda _m$ the largest and the smallest eigenvalues of matrix $P_k^{-1/2}((\mu _k-1)AA^T+2 A (I_n \otimes U_kU_k^T) A^T) P_k^{-1/2}$, respectively. From (4.19) we deduce

$$\begin{aligned} \lambda _{\min }(P_k) \ge \mu _k (\sigma _{\min }(A))^2 \end{aligned}$$

and

$$\begin{aligned} \lambda _{\max }(A (I_n \otimes U_kU_k^T) A^T) \le (\sigma _{max}(A))^2 \lambda _{\max } (U_kU_k^T). \end{aligned}$$

Then, using the Weyl inequality we obtain

$$\begin{aligned} \lambda _M \le \frac{(\sigma _{max}(A))^2(\mu _k + \lambda _{\max } (U_kU_k^T))}{\mu _k (\sigma _{\min }(A))^2}. \end{aligned}$$

Moreover,

$$\begin{aligned} \lambda _{\min }(P_k^{-1/2} AA^TP_k^{-1/2})=\frac{1}{\lambda _{\max }(P_k^{1/2} (AA^T)^{-1}P_k^{1/2})} \ge \frac{(\sigma _{\min } (A))^2}{\Vert P_k\Vert _2}. \end{aligned}$$

Then, noting that $\Vert P_k\Vert _2 \le (\sigma _{\max } (A))^2( \mu _k +(\lambda _{\max }(U_kU_k^T))^2)$, we have

$$\begin{aligned} \lambda _m \ge \frac{ (\mu _k-1) (\sigma _{\min }(A))^2}{(\sigma _{\max } (A))^2 (\mu _k+(\lambda _{\max }(U_kU_k^T))^2)}. \end{aligned}$$

Consequently, the eigenvalues of the preconditioned matrix $P_k^{-1/2}(A (I \otimes X^2) A^T)P_k^{-1/2}$ belong to the interval $(1+\xi _1,1+\xi _2)$, and the theorem follows. $\square$

Note that from the result above, as $\mu _k$ approaches zero, the minimum eigenvalue of the preconditioned matrix goes to one and the maximum remains bounded.

The application of $P_k$ to a vector *d*, needed at each CG iteration, can be performed through the solution of the $(m+nq) \times (m+nq)$ sparse augmented system:

4.22 $$\begin{aligned} \begin{bmatrix} \mu _k AA^T &{} Z_k\\ Z_k^T &{} -I_{nr} \end{bmatrix} \begin{bmatrix} u \\ v \end{bmatrix} = \begin{bmatrix} d \\ 0 \end{bmatrix}, \end{aligned}$$

where if $Z_k$ is given by (4.20) $q=n$, while $q=r$ in case (4.21). In order to recover the vector $u=P_k^{-1}d$, we can solve the linear system

4.23 $$\begin{aligned} (I_{nr} + Z_k^T ( \mu _kA A^T )^{-1} Z_k) v = Z_k^T ( \mu _k A A^T )^{-1} d, \end{aligned}$$

and compute *u* as follows

$$\begin{aligned} u = ( \mu _k A A^T )^{-1} (d - Z_k v). \end{aligned}$$

This process involves the inversion of $A A^T$ which can be done once at the beginning of the iterative process, and the solution of a linear system with matrix

$$\begin{aligned} E_k = I+Z_k^T(\mu _k AA^T)^{-1}Z_k. \end{aligned}$$

Note that $E_k$ has dimension $n^2 \times n^2$ in case of choice (4.20) and dimension $nr \times nr$ in case of choice (4.21). Then, its inversion is impractical in case (4.20). On the other hand, using (4.21) we can approximately solve (4.23) using a CG-like solver.

At this regard, observe that the entries of $E_k$ decrease when far away from the main diagonal and $E_k$ can be preconditioned by its block-diagonal part, that is by

4.24 $$\begin{aligned} M_k = I_{nr} + {{\mathcal {B}}} (Z_k^T ( \mu _k A A^T )^{-1} Z_k), \end{aligned}$$

where ${{\mathcal {B}}}$ is the operator that extracts from a matrix $nr \times nr$ its block diagonal part with *n* diagonal blocks of size $r\times r$.

## SDP Reformulation of Matrix Completion Problems

We consider the problem of recovering a low-rank data matrix $B \in {\mathbb {R}}^{{\hat{n}} \times {\hat{n}}}$ from a sampling of its entries [13], that is the so called *matrix completion* problem. The problem can be stated as

5.1 $$\begin{aligned} \begin{array}{ll} \min &{} rank({\bar{X}}) \\ [0.2cm] \text{ s.t. } &{} {\bar{X}}_{\Omega } = B_{\Omega }, \end{array} \end{aligned}$$

where $\Omega$ is the set of locations corresponding to the observed entries of *B* and the equality is meant element-wise, that is $X_{s,t} = B_{s,t}, \text{ for } \text{ all } (s,t) \in \Omega$. Let *m* be the cardinality of $\Omega$ and *r* be the rank of *B*.

A popular convex relaxation of the problem [13] consists in finding the minimum nuclear norm of ${\bar{X}}$ that satisfies the linear constraints in (5.1), that is, solving the following heuristic optimization

5.2 $$\begin{aligned} \begin{array}{ll} \min &{} \Vert {\bar{X}} \Vert _* \\ [0.2cm] \text{ s.t. } &{} {\bar{X}}_{\Omega } = B_{\Omega }, \end{array} \end{aligned}$$

where the nuclear norm $\Vert \cdot \Vert _*$ of ${\bar{X}}$ is defined as the sum of its singular values.

Candès and Recht proved in [13] that if $\Omega$ is sampled uniformly at random among all subset of cardinality *m* then with large probability, the unique solution to (5.2) is exactly *B*, provided that the number of samples obeys $m \ge C {\hat{n}}^{5/4} r \log {\hat{n}}$, for some positive numerical constant *C*. In other words, problem (5.2) is “formally equivalent” to problem (5.1). Let

5.3 $$\begin{aligned} X=\begin{bmatrix} W_1 &{} {\bar{X}} \\ {\bar{X}}^T &{} W_2 \end{bmatrix}, \end{aligned}$$

where ${\bar{X}} \in {\mathbb {R}}^{{\hat{n}}\times {\hat{n}}}$ is the matrix to be recovered and $W_1, W_2 \in S{\mathbb {R}}^{{\hat{n}}\times {\hat{n}}}$. Then problem (5.2) can be stated as an SDP of the form (1.1) as follows

5.4 $$\begin{aligned} \begin{array}{ll} \min &{} \frac{1}{2} I \bullet X \\ [0.2cm] \text{ s.t. } &{} \begin{bmatrix} 0 &{} \Theta _{st} \\ \Theta _{st}^T &{} 0 \end{bmatrix} \bullet X = B_{(s,t)}, \quad (s,t) \in \Omega \\ [0.5cm] &{} X\succeq 0, \end{array} \end{aligned}$$

where for each $(s,t) \in \Omega$ the matrix $\Theta _{st} \in {\mathbb {R}}^{{\hat{n}} \times {\hat{n}}}$ is defined element-wise for $k,l = 1, \dots , {\hat{n}}$ as

$$\begin{aligned} (\Theta _{st} )_{kl}= \left\{ \begin{array}{ll} 1/2 &{} \text{ if } (k,l) = (s,t) \\ 0 &{} \text{ otherwise, } \end{array} \right. \end{aligned}$$

see [39]. We observe that primal variable *X* takes the form (5.3) with $n= 2{\hat{n}}$, the symmetric matrix *C* in the objective of (1.1) is a scaled identity matrix of dimension $n \times n$. The vector $b\in {\mathbb {R}}^m$ is defined by the known elements of *B* and, for $i=1,\ldots ,m$, each constraint matrix $A_i$, corresponds to the known elements of *B* stored in $b_i$. Matrices $A_i$ have a very special structure that yields nice properties in the packed matrix *A*. Since every constraint matrix has merely two nonzero entries the resulting matrix *A* has 2*m* nonzero elements and its density is equal to $2n^{-2}$. Moreover, $AA^T=\frac{1}{2}I_m$ and $\Vert {{\mathcal {A}}} (I_n)\Vert _2 = 0$.

We now discuss the relationship between a rank *r* solution ${\bar{X}}$ of problem (5.2) and a rank *r* solution *X* of problem (5.4).

### Proposition 5.1

If *X* of the form $$\begin{bmatrix} W_1 &{} {\bar{X}} \\ {\bar{X}}^T &{} W_2 \end{bmatrix}$$ with ${\bar{X}} \in {\mathbb {R}}^{{\hat{n}}\times {\hat{n}}}$ and $W_1, W_2 \in S{\mathbb {R}}^{{\hat{n}}\times {\hat{n}}}$ has rank *r*, then ${\bar{X}}$ has rank *r*. Vice-versa, if ${\bar{X}}$ has rank *r* with ${\bar{X}} \in {\mathbb {R}}^{{\hat{n}}\times {\hat{n}}}$, then there exist $W_1, W_2 \in S{\mathbb {R}}^{{\hat{n}}\times {\hat{n}}}$ such that $$\begin{bmatrix} W_1 &{} {\bar{X}} \\ {\bar{X}}^T &{} W_2 \end{bmatrix}$$ has rank *r*.

### Proof

Let $X = Q \Sigma Q^T$ with $Q \in {\mathbb {R}}^{2 {\hat{n}}\times r}$ and $\Sigma = {\mathbb {R}}^{r \times r}$ be the singular value decomposition (SVD) of *X*. Let *Q* be partitioned by $$Q = \begin{bmatrix} Q_1 \\ Q_2 \end{bmatrix}$$ with $Q_1, Q_2 \in {\mathbb {R}}^{ {\hat{n}}\times r}$. Then

$$\begin{aligned} X = \begin{bmatrix} Q_1 \\ Q_2 \end{bmatrix} \Sigma \begin{bmatrix} Q_1^T&Q_2^T \end{bmatrix} = \begin{bmatrix} Q_1 \Sigma Q_1^T &{} Q_1 \Sigma Q_2^T \\ Q_2 \Sigma Q_1^T &{} Q_2 \Sigma Q_2^T \end{bmatrix}, \end{aligned}$$

that is ${\bar{X}}=Q_1\Sigma Q_2^T$ has rank *r*.

To prove the second part of the proposition, let ${\bar{X}} = Q \Sigma V^T$ with $Q, V\in {\mathbb {R}}^{ {\hat{n}}\times r}$ and $\Sigma = {\mathbb {R}}^{r\times r}$ be the SVD factorization of ${\bar{X}}$. We get the proposition by defining $W_1 = Q \Sigma Q^T$ and $W_2 = V \Sigma V^T$ and obtaining $$X=\begin{bmatrix} Q\\ V \end{bmatrix} \Sigma \begin{bmatrix} Q^T&V^T \end{bmatrix} . $$ $\square$

### Corollary 5.2

Let *X* be structured as $$\begin{bmatrix} W_1 &{} {\bar{X}} \\ {\bar{X}}^T &{} W_2 \end{bmatrix}$$ with ${\bar{X}} \in {\mathbb {R}}^{{\hat{n}}\times {\hat{n}}}$ and $W_1, W_2 \in S{\mathbb {R}}^{{\hat{n}}\times {\hat{n}}}$. Assume that *X* has the form

$$\begin{aligned} X = UU^T + \mu I, \end{aligned}$$

with $U \in {\mathbb {R}}^{n\times r}$ full column rank and $\mu \in {\mathbb {R}}$, then ${\bar{X}}$ has rank r.

### Proposition 5.3

If *X* is a rank *r* solution of (5.4), then ${\bar{X}}$ is a rank *r* solution of (5.2). Vice-versa, if ${\bar{X}}$ is a rank *r* solution of (5.2), then (5.4) admits a rank *r* solution.

### Proof

The first statement follows from the equivalence between problems (5.4) and (5.2) [19, Lemma 1].

Let ${\bar{X}}$ be a rank *r* optimal solution of (5.2), $t^* = \Vert {\bar{X}}\Vert _*$ and $Q \Sigma V^T$, with $Q, V\in {\mathbb {R}}^{ {\hat{n}}\times r}$ and $\Sigma \in {\mathbb {R}}^{r\times r}$, be the SVD decomposition of ${\bar{X}}$. Let us define $$X = \begin{bmatrix} W_1 &{} {\bar{X}} \\ {\bar{X}}^T &{} W_2 \end{bmatrix}$$ with $W_1 = Q \Sigma Q^T$ and $W_2 = V \Sigma V^T$. Then *X* solves (5.4). In fact, *X* is positive semidefinite and $\frac{1}{2}I \bullet X = \frac{1}{2}(Trace(W_1)+Trace(W_2)) = \Vert {\bar{X}}\Vert _* = t^*$. This implies that $t^*$ is the optimal value of (5.4). In fact, if we had *Y* such that

$$\begin{aligned} \begin{bmatrix} 0 &{} \Theta _{st} \\ \Theta _{st}^T &{} 0 \end{bmatrix} \bullet Y = B_{(s,t)}, \quad (s,t) \in \Omega \quad \quad Y\succeq 0 \end{aligned}$$

and $\frac{1}{2}I \bullet Y \le t^\star$, then by [19, Lemma 1] there would exist ${\bar{Y}}$ such that $\Vert {\bar{Y}}\Vert _* < t^*$, that is $\Vert {\bar{Y}}\Vert _* <\Vert {\bar{X}}\Vert _*=t^*$. This is a contradiction as we assumed that $t^*$ is the optimal value of (5.2). $\square$

**Remark.** Assuming that a rank *r* solution to (5.2) exists, the above analysis justifies the application of our algorithm to search for a rank *r* solution of the SDP reformulation (5.4) of (5.2). We also observe that at each iteration our algorithm computes an approximation $X_k$ of the form $X_k=U_kU_k^T+\mu _k I_n$ with $U_k\in {\mathbb {R}}^{n\times r}$ and $\mu _k>0$. Then, if at each iteration $U_k$ is full column rank, by Corollary 5.2, it follows that we generate a sequence $\{{\bar{X}}_k\}$ such that ${\bar{X}}_k$ has exactly rank *r* at each iteration *k* and it approaches a solution of (5.2).

Finally, let us observe that $m<{{\hat{n}}}^2=n^2/4$ and $nnz(A)=2m<n^2/2$. Then, by the analysis carried out in Sect. 4.1 each evaluation of the gradient of $\phi _{\mu _k}$ amounts to $O(n^2r)$ flops and assuming to use a first-order method at each iteration to compute $(U_k,{\bar{y}}_k)$, in the worst-case each iteration of our method requires $O(\mu _k^{-2}n^2r)$ flops.

## Numerical Experiments on Matrix Completion Problems

We consider an application to matrix completion problems by solving (5.4) with our relaxed Interior Point algorithm for Low-Rank SDPs (IPLR), described in Algorithm 2. IPLR has been implemented using Matlab (R2018b) and all experiments have been carried out on Intel Core i5 CPU 1.3 GHz with 8 GB RAM. Parameters in Algorithm 2 have been chosen as follows:

$$\begin{aligned} \mu _0 = 1,\ \sigma = 0.5,\ \eta _1 = 0.9,\ \eta _2 = \sqrt{n}, \end{aligned}$$

while the starting dual feasible approximation has been chosen as $y_0=0, S_0=\frac{1}{2}I_n$ and $U_0$ is defined by the first *r* columns of the identity matrix $I_n$.

We considered two implementations of IPLR which differ with the strategy used to find a minimizer of $\phi _{\mu _k}(U,y)$ (Line 3 of Algorithm 2).

Let IPLR-GS denote the implementation of IPLR where the Gauss–Seidel strategy described in Algorithm 3 is used to find a minimizer of $\phi _{\mu _k}(U,y)$. We impose a maximum number of 5 $\ell$-iterations and use the (possibly) preconditioned conjugate gradient method to solve the linear systems (4.13) and (4.14). We set a maximum of 100 CG iterations and the tolerance $10^{-6}$ on the relative residual of the linear systems. System (4.13) is solved with unpreconditioned CG. Regarding (4.14), for the sake of comparison, we report in the next section statistics using unpreconditioned CG and CG employing the preconditioner defined by (4.19) and (4.21). In this latter case the action of the preconditioner has been implemented through the augmented system (4.22), following the procedure outlined at the end of Sect. 5. The linear system (4.23) has been solved by preconditioned CG, with preconditioner (4.24) allowing a maximum of 100 CG iterations and using a tolerance $10^{-8}$. In fact, the linear system (4.14) along the IPLR iterations becomes ill-conditioned and the application of the preconditioner needs to be performed with high accuracy. We will refer to the resulting method as IPLR-GS_P.

As an alternative implementation to IPLR-GS, we considered the use of a first-order approach to perform the minimization at Line 3 of Algorithm 2. We implemented the Barzilai-Borwein method [3, 38] with a non-monotone line-search following [17, Algorithm 1] and using parameter values as suggested therein. The Barzilai-Borwein method iterates until $\Vert \nabla \phi _{\mu _k}(U_k,y_k) \Vert \le \min (10^{-3}, \mu _k)$ or a maximum of 300 iterations is reached. We refer to the resulting implementation as IPLR-BB.

The recent literature for the solution of matrix completion problems is very rich and there exist many algorithms finely tailored for such problems, see e.g. [11, 14, 28, 33, 35, 37, 42, 45] just to name a few. Among these, we chose the OptSpace algorithm proposed in [28, 29] as a reference algorithm in the forthcoming tests. In fact, OptSpace compares favourably [29] with the state-of-art solvers such as SVT [11], ADMiRA [33] and FPCA [37] and its Matlab implementation is publicly available online.^1^OptSpace is a first-order algorithm. Assuming the known solution rank *r*, it first generates a good starting guess by computing the truncated SVD (of rank *r*) of a suitable sparsification of the available data $B_{\Omega }$ and then uses a gradient-type procedure in order to minimize the error $\Vert B-Q\Sigma V^T\Vert _F$ where $Q,\Sigma , V$ are the SVD factors of the current solution approximation. Since *Q* and *V* are orthonormal matrices, the minimization in these variables is performed over the Cartesian product of Grassmann manifolds, while minimization in $\Sigma$ is computed exactly in $\mathbb {R}^{r\times r}$. In [29], OptSpace has been equipped with two strategies to accommodate the unknown solution rank: the first strategy aims at finding a split in the eigenvalue distribution of the sparsified (“trimmed”) matrix and on accurate approximation of its singular values and the corresponding singular vectors; the second strategy starts from the singular vectors associated with the largest singular value and incrementally searches for the next singular vectors. The latter strategy yields the so called Incremental OptSpace variant, proposed to handle ill-conditioned problems whenever an accurate approximation of the singular vector corresponding to the smallest singular value is not possible and the former strategy fails.

Matlab implementations of OptSpace and Incremental OptSpace have been employed in the next sections. We used default parameters except for the maximum number of iterations. The default value is 50 and, as reported in the next sections, it was occasionally increased to improve accuracy in the computed solution.

We perform two sets of experiments: the first aims at validating the proposed algorithms and is carried out on randomly generated problems; the second is an application of the new algorithms to real data sets.

### Tests on Random Matrices

As it is a common practice for a preliminary assessment of new methods, in this section we report on the performance of our proposed IPLR algorithm on matrices which have been randomly generated. We have generated random matrices both with noise and without noise, random nearly low-rank matrices and random mildly ill-conditioned matrices with and without noise. For the last class of matrices, which we expect to mimic reasonably well the practical problems, we also report the solution statistics obtained with OptSpace.

We have generated ${\hat{n}} \times {\hat{n}}$ matrices of rank *r* by sampling two ${\hat{n}} \times r$ factors $B_L$ and $B_R$ independently, each having independently and identically distributed Gaussian entries, and setting $B = B_L B_R$. The set of observed entries $\Omega$ is sampled uniformly at random among all sets of cardinality *m*. The matrix *B* is declared recovered if the (2,1) block ${\bar{X}}$ extracted from the solution *X* of (5.4), satisfies

6.1 $$\begin{aligned} \Vert {\bar{X}} - B\Vert _F / \Vert B\Vert _F < 10^{-3}, \end{aligned}$$

see [13].

Given *r*, we chose *m* by setting $m = c r(2{\hat{n}}-r)$, ${\hat{n}}=600,700,800,900,1000$. We used $c=0.01 {\hat{n}}+4$. These corresponding values of *m* are much lower than the theoretical bound provided by [13] and recalled in Sect. 5, but in our experiments they were sufficient to recover the sought matrix by IPLR.

In our experiments, the accuracy level in the matrix recovery in (6.1) is always achieved by setting $\epsilon = 10^{-4}$ in Algorithm 2.

In the forthcoming tables we report: dimensions *n* and *m* of the resulting SDPs and target rank *r* of the matrix to be recovered; being *X* and *S* the computed solution, the final primal infeasibility $\Vert {{\mathcal {A}}}(X)-b\Vert$, the complementarity gap $\Vert XS-\mu I\Vert _F$, the error in the solution of the matrix completion problem ${{\mathcal {E}}}= \Vert {\bar{X}} - B\Vert _F /\Vert B\Vert _F$, the overall cpu time in seconds.

**Table 2:** IPLR-GS on random matrices

rank/*n*/*m*	IPLR-GS				
	$\Vert {{\mathcal {A}}}(X)-b\Vert$	$\Vert XS-\mu I\Vert _F$	$\lambda _{\min }(S)$	${\mathcal {E}}$	cpu/cpu_P
3/1200/35910	4E−04	1E−03	4E−08	2E−06	229/**110**
4/1200/47840	2E−04	1E−03	4E−08	9E−07	173/**99**
5/1200/59750	4E−05	1E−03	4E−08	1E−07	156/**104**
6/1200/71640	2E−06	1E−03	4E−08	5E−09	219/**201**
7/1200/83510	5E−07	1E−03	4E−08	9E−10	**164**/199
8/1200/95360	5E−08	1E−03	4E−08	8E−11	**152**/228
3/1400/46101	3E−04	1E−03	4E−08	1E−06	362/**148**
4/1400/61424	1E−04	1E−03	4E−08	8E−07	352/**175**
5/1400/76725	5E−05	1E−03	4E−08	1E−07	205/**151**
6/1400/92004	7E−06	1E−03	4E−08	1E−08	223/**199**
7/1400/107261	2E−07	1E−03	3E−08	4E−10	**214**/239
8/1400/122496	2E−08	1E−03	3E−08	3E−11	**234**/329
3/1600/57492	3E−04	1E−03	3E−08	1E−06	330/**168**
4/1600/76608	1E−04	1E−03	3E−08	4E−07	387/**174**
5/1600/95700	4E−05	1E−03	3E−08	9E−08	433/**235**
6/1600/114768	1E−06	1E−03	3E−08	2E−09	316/**226**
7/1600/133812	2E−07	1E−03	3E−08	2E−10	393/**331**
8/1600/152832	4E−08	1E−03	3E−08	5E−11	**334**/370
3/1800/64692	4E−04	1E−03	3E−08	2E−06	566/**259**
4/1800/86208	3E−04	1E−03	3E−08	7E−07	506/**231**
5/1800/107700	4E−05	1E−03	3E−08	1E−07	465/**270**
6/1800/129168	1E−05	1E−03	3E−08	6E−08	586/**364**
7/1800/150612	8E−07	1E−03	3E−08	3E−9	606/**462**
8/1800/172032	4E−07	1E−03	3E−08	1E−9	831/**795**
3/2000/83874	3E−04	1E−03	2E−08	1E−06	599/**400**
4/2000/111776	3E−04	1E−03	2E−08	7E−07	544/**365**
5/2000/139650	1E−05	1E−03	2E−08	3E−08	783/**512**
6/2000/167496	2E−06	1E−03	2E−08	3E−09	601/**485**
7/2000/195314	2E−07	1E−03	2E−08	2E−10	657/**594**
8/2000/223104	2E−08	1E−03	2E−08	4E−11	**627**/669

In Tables 2 and 3 we report statistics of IPLR-GS and IPLR-BB, respectively. We choose as a starting rank *r* the rank of the matrix *B* to be recovered. In the last column of Table 2 we report both the overall cpu time of IPLR-GS without preconditioner (cpu) and with preconditioner (cpu_P) in the solution of (4.14). The lowest computational time for each problem is indicated in bold.

**Table 3:** IPLR-BB on random matrices

rank/*n*/*m*	IPLR-BB				
	$\Vert {{\mathcal {A}}}(X)-b\Vert$	$\Vert XS-\mu I\Vert _F$	$\lambda _{\min }(S)$	${{\mathcal {E}}}$	cpu
3/1200/35910	4E−06	1E−03	4E−08	2E−08	223
4/1200/47840	1E−05	1E−03	4E−08	3E−08	186
5/1200/59750	6E−06	1E−03	4E−08	2E−08	235
6/1200/71640	8E−06	1E−03	4E−08	1E−08	242
7/1200/83510	4E−06	1E−03	4E−08	9E−09	237
8/1200/95360	6E−06	1E−03	4E−08	1E−08	223
3/1400/46101	8E−06	1E−03	4E−08	3E−08	402
4/1400/61424	2E−06	1E−03	4E−08	8E−08	402
5/1400/76725	6E−06	1E−03	4E−08	1E−08	332
6/1400/92004	4E−06	1E−03	3E−08	9E−09	403
7/1400/107261	2E−06	1E−03	3E−08	4E−09	361
8/1400/122496	2E−06	1E−03	3E−08	6E−09	386
3/1600/57492	2E−04	1E−03	3E−08	6E−09	557
4/1600/76608	4E−06	1E−03	3E−08	8E−09	620
5/1600/95700	2E−06	1E−03	3E−08	5E−08	506
6/1600/114768	2E−06	1E−03	3E−08	3E−09	477
7/1600/133812	4E−06	1E−03	3E−08	5E−09	571
8/1600/152832	4E−07	1E−03	3E−08	5E−10	600
3/1800/64692	9E−06	1E−03	3E−08	6E−08	573
4/1800/86208	8E−06	1E−03	3E−08	4E−08	906
5/1800/107700	4E−06	1E−03	3E−08	1E−08	784
6/1800/129168	2E−06	1E−03	3E−08	6E−09	686
7/1800/150612	3E−06	1E−03	3E−08	1E−8	625
8/1800/172032	4E−07	1E−03	3E−08	1E−8	862
3/2000/83874	7E−06	1E−03	3E−08	3E−08	900
4/2000/111776	4E−07	1E−03	3E−08	9E−10	1000
5/2000/139650	4E−06	1E−03	3E−08	1E−08	921
6/2000/167496	7E−06	1E−03	2E−08	1E−08	900
7/2000/195314	3E−07	1E−03	2E−08	3E−10	1000
8/2000/223104	4E−08	1E−03	2E−08	3E−09	931

As a first comment, we verified that Assumption 1 in Sect. 2 holds in our experiments. In fact, the method manages to preserve positive definiteness of the dual variable and $\alpha _k<1$ is taken only in the early stage of the iterative process.

Secondly, we observe that both IPLR-GS and IPLR-BB provide an approximation to the solution of the sought rank; in some runs the updating procedure increases the rank, but at the subsequent iteration the downdating strategy is activated and the procedure comes back to the starting rank *r*. Moreover, IPLR-GS is overall less expensive than IPLR-BB in terms of cpu time, in particular as *n* and *m* increase. In fact, the cost of the linear algebra in the IPLR-GS framework is contained as one/two inner Gauss–Seidel iterations are performed at each outer IPLR-GS iteration except for the very few initial ones where up to five inner Gauss–Seidel iterations are needed. To give more details of the computational cost of both methods, in Table 4 we report some statistics of IPLR-GS and IPLR-BB for ${\hat{n}}=900$, $r=3$ and 8. More precisely we report the average number of inner Gauss–Seidel iterations (avr_GS) and the average number of unpreconditioned CG iterations in the solution of (4.13) (avr_CG_1) and (4.14) (avr_CG_2) for IPLR-GS and the average number of BB iterations for IPLR-BB (avr_BB). We notice that the solution of SDP problems becomes more demanding as the rank increases, but both the number of BB iterations and the number of CG iterations are reasonable.

**Table 4:** Statistics of IPLR-GS and IPLR-BB on random matrix ${\hat{n}}=900$, $r=3$ and 8

rank/*n*/*m*	IPLR-GS			IPLR-BB
	avr_GS	avr_CG_1	avr_CG_2	avr_BB
3/1800/64692	2.1	15.3	24.2	68
8/1800/172032	2.0	19.5	42.2	88

**Fig. 2:**
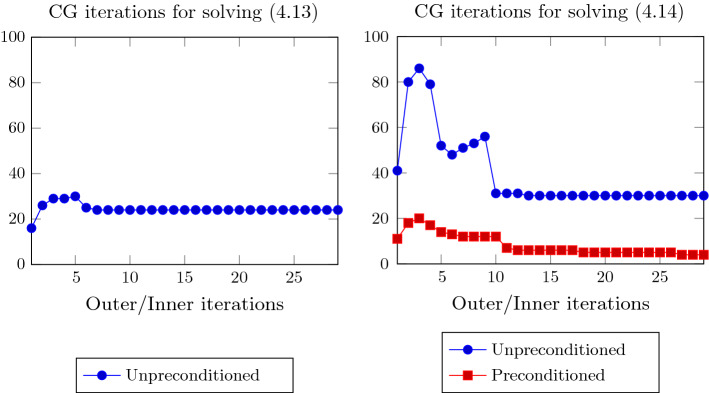
CG iterations for solving systems (4.13) with IPLR-GS (left) and CG iterations for solving systems (4.14) with IPLR-GS and IPLR-GS_P (right). Data: ${\hat{n}}=100$, $r=4$

To provide an insight into the linear algebra phase, in Fig. 1 we plot the minimum nonzero eigenvalue and the maximum eigenvalue of the coefficient matrix of (4.13), i.e. $Q_k^T (A^TA + (S_k^2 \otimes I_n)) Q_k$. We remark that the matrix depends both on the outer iteration *k* and on the inner Gauss–Seidel iteration $\ell$ and we dropped the index $\ell$ to simplify the notation. Eigenvalues are plotted against the inner/outer iterations, for ${\hat{n}}=100$, $r=4$ and IPLR-GS continues until $\mu _k<10^{-7}$. In this run only one inner iteration is performed at each outer iteration except for the first outer iteration. We also plot in the left picture of Fig. 2 the number of CG iterations versus inner/outer iterations. The figures show that the condition number of $Q_k$ and the overall behaviour of CG do not depend on $\mu _k$. Moreover, Table 4 shows that unpreconditioned CG is able to reduce the relative residual below $10^{-6}$ in a low number of iterations even in the solution of larger problems and higher rank. These considerations motivate our choice of solving (4.13) without employing any preconditioner.

**Fig. 3:**
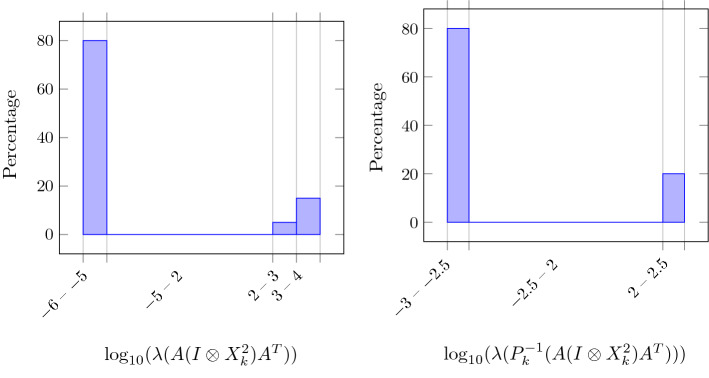
Eigenvalue distribution of $A (I \otimes X_k^2) A^T$ (left) and $P_k^{-1}(A (I \otimes X_k^2) A^T)$ (right) at the first inner iteration of outer IPLR-GS iteration corresponding to $\mu _k\approx 1.9 e-3$ (semilog scale). Data: ${\hat{n}}=100$, $r=4$

We now discuss the effectiveness of the preconditioner $P_k$ given in (4.19), with $Z_k$ given in (4.21), in the solution of (4.14). Considering ${\hat{n}}=100$, $r=4$, in Fig. 3 we plot the eigenvalue distribution (in percentage) of $A (I \otimes X_k^2) A^T$ and $P_k^{-1}(A (I \otimes X_k^2) A^T)$ at the first inner iteration of outer IPLR-GS iteration corresponding to $\mu _k \approx 1.9e\!-\!3$. We again drop the index $\ell$. We can observe that the condition number of the preconditioned matrix is about 1.3*e*5, and it is significantly smaller than the condition number of the original matrix (about 3.3*e*10). The preconditioner succeeded both in pushing the smallest eigenvalue away from zero and in reducing the largest eigenvalue. However, CG converges in a reasonable number of iterations even in the unpreconditioned case, despite the large condition number. In particular, we can observe in the right picture of Fig. 2 that preconditioned CG takes less than five iterations in the last stages of IPLR-GS and that the most effort is made in the initial stage of the IPLR-GS method; in this phase the preconditioner is really effective in reducing the number of CG iterations. These considerations remain true even for larger values of ${\hat{n}}$ and *r* as it is shown in Table 4.

**Fig. 4:**
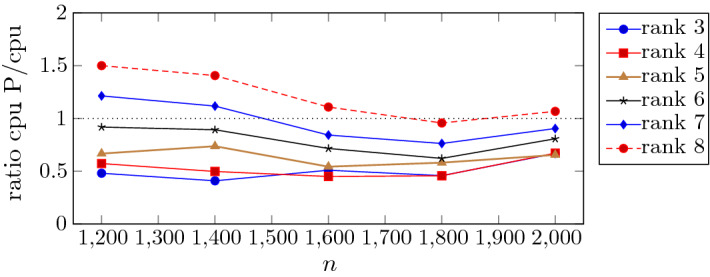
The ratio cpu_P/cpu as a funcion of dimension *n* and of the rank (data extracted from Table 2)

Focusing on the computational cost of the preconditioner’s application, we can observe from the cpu times reported in Table 2, that for $r=3,4,5$ the employment of the preconditioner produces a great benefit, with savings that vary from $20\%$ to $50\%$. Then, the overhead associated to the construction and application of the preconditioner is more than compensated by the gains in the number of CG iterations. The cost of application of the preconditioner increases with *r* as the dimension of the diagonal blocks of $M_k$ in (4.24) increases with *r*. Then, for small value of ${\hat{n}}$ and $r=6,7,8$ unpreconditioned CG is preferable, while for larger value of ${\hat{n}}$ the preconditioner is effective in reducing the overall computational time for $r\le 7$. This behaviour is summarized in Fig. 4 where we plot the ratio cpu_P/cpu with respect to dimension *n* and rank (from 3 to 8).

In the approach proposed in this paper the primal feasibility is gradually reached, hence it is also possible to handle data $B_{\Omega }$ corrupted by noise. To test how the method behaves in such situations we set ${\hat{B}}_{(s,t)} = B_{(s,t)} + \eta RD_{(s,t)}$ for any $(s,t) \in \Omega$, where $RD_{(s,t)}$ is a random scalar drawn from the standard normal distribution, generated by the Matlab function randn; $\eta >0$ is the level of noise. Then, we solved problem (5.4) using the corrupted data ${\hat{B}}_{(s,t)}$ to form the vector *b*. Note that, in this case $\Vert {{\mathcal {A}}}(B)-b\Vert _2\approx \eta \sqrt{m}$. In order to take into account the presence of noise we set $\epsilon = \max (10^{-4},10^{-1} \eta )$ in Algorithm 2.

Results of these runs are collected in Table 5 where we considered $\eta = 10^{-1}$ and started with the target rank *r*. In Table 5 we also report

$$\begin{aligned} RMSE= \Vert {\bar{X}} - B\Vert _F /{\hat{n}}, \end{aligned}$$

that is the root-mean squared error per entry. Note that the root-mean error per entry in data $B_{\Omega }$ is of the order of the noise level $10^{-1}$, as well as $\Vert {{\mathcal {A}}}(B)-b\Vert _2/\sqrt{m}$. Then, we claim to recover the matrix with acceptable accuracy, corresponding to an average error smaller than the level of noise.

**Table 5:** IPLR-GS_P on noisy matrices (noise level $\eta = 10^{-1}$)

rank/*n*/*m*	IPLR-GS_P				
	$\Vert {{\mathcal {A}}}(X)-b\Vert$	$\Vert XS-\mu I\Vert _F$	$\lambda _{\min }(S)$	$\Vert {\bar{X}} - B\Vert _F /{\hat{n}}$	cpu
4/1200/47840	2E01	1E−01	6E−06	3E−02	67
6/1200/71640	2E01	1E−01	6E−06	3E−02	128
8/1200/95360	3E01	1E−01	5E−06	3E−02	182
4/1600/76608	3E01	2E−01	4E−06	3E−02	178
6/1600/114768	3E01	2E−01	4E−06	3E−02	224
8/1600/152832	4E01	2E−01	4E−06	3E−02	358
4/2000/111776	3E01	2E−01	4E−06	3E−02	259
6/2000/167496	4E01	2E−01	4E−06	3E−02	373
8/2000/223104	4E01	2E−01	4E−06	3E−02	543

### Mildly Ill-Conditioned Problems

**Fig. 5:**
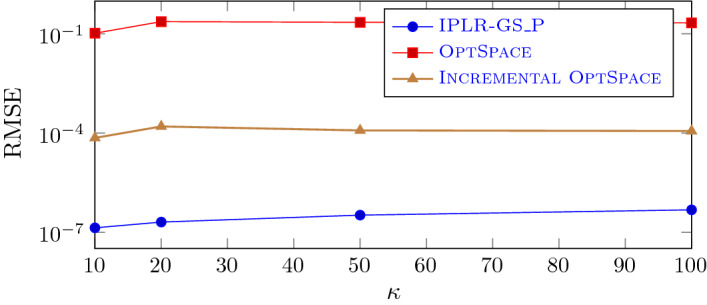
IPLR-GS_P, OptSpace and Incremental OptSpace on mildly ill-conditioned matrices (semilog scale ${\hat{n}}=600$, $r=6$, $n=1200$, $m=47840$)

In this subsection we compare the performance of IPLR_GS_P, OptSpace and Incremental OptSpace on mildly ill-conditioned problems with exact and noisy observations. We first consider exact observation and vary the condition number of the matrix that has to be recovered $\kappa$. We fixed ${\hat{n}}=600$ and $r=6$ and, following [29], generated random matrices with a prescribed condition number $\kappa$ and rank *r* as follows. Given a random matrix *B* generated as in the previous subsection, let $Q\Sigma V^T$ be its SVD decomposition and ${\tilde{Q}}$ and ${\tilde{V}}$ be the matrices formed by the first *r* columns of *Q* and *V*, respectively. Then, we formed the matrix ${\hat{B}}$ that has to be recovered as ${\hat{B}}={\tilde{Q}} {\tilde{\Sigma }} {\tilde{V}}^T$, where ${\tilde{\Sigma }}$ is a $r\times r$ diagonal matrix with diagonal entries equally spaced between ${\hat{n}}$ and ${\hat{n}}/\kappa$. In Fig. 5 we plot the RMSE value against the condition number for all the three solvers considered, using the $13\%$ of the observations. We can observe, as noticed in [29], that OptSpace does not manage to recover mildly ill-conditioned matrices while Incremental OptSpace improves significantly over OptSpace. According to [29], the convergence difficulties of OptSpace on these tests have to be ascribed to the singular value decomposition of the trimmed matrix needed in Step 3 of OptSpace. In fact, the singular vector corresponding to the smallest singular value cannot be approximated with enough accuracy. On the other hand, our approach is more accurate than Incremental OptSpace and its behaviour only slightly deteriorates as $\kappa$ increases.

**Fig. 6:**
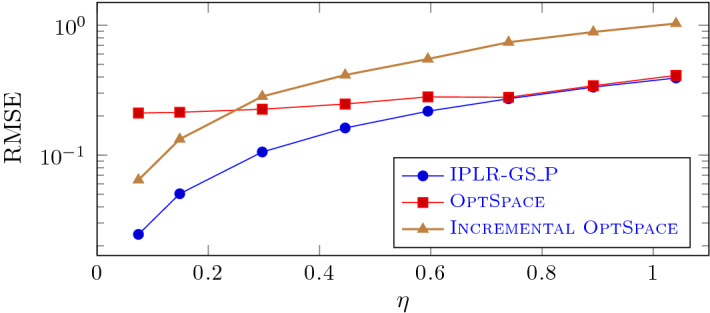
IPLR-GS_P, OptSpace and Incremental OptSpace on noisy and mildly ill-conditioned matrices (semilog scale, $\kappa =200$, ${\hat{n}}=600$, $r=6$, $n=1200$, $m=71,640$)

Now, let us focus on the case of noisy observations. We first fixed $\kappa =200$ and varied the noise level. In Fig. 6 we plot the RMSE value against the noise level for all the three solvers considered, using the $20\%$ of observations. Also in this case IPLR-GS_P is able to recover the matrix ${\hat{B}}$ with acceptable accuracy, corresponding to an average error smaller than the level of noise, and outperforms both OptSpace variants when the noise level is below 0.8. In fact, OptSpace managed to recover ${\hat{B}}$ only with a corresponding *RMSE* of the order of $10^{-1}$ for any tested noise level, consistent only with the larger noise level tested.

**Fig. 7:**
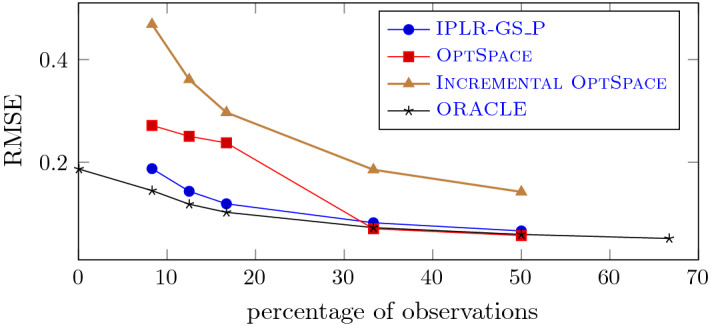
IPLR-GS_P, OptSpace and Incremental OptSpace on noisy and mildly ill-conditioned matrices, varying the percentage of observations ($\kappa =100$, ${\hat{n}}=600$, $r=6$, $n=1200$, $\eta =0.3$)

In order to get a better insight into the behaviour of the method on mildly ill-conditioned and noisy problems, we fixed $\kappa =100$, noise level $\eta =0.3$ and varied the percentage of known entries from 8.3% to 50%, namely we set $m=30,000, 45,000,$ 60, 000, 120, 000, 180, 000. In Fig. 7 the value of *RMSE* is plotted against the percentage of known entries. The oracle error value $RMSE_{or}=\eta \sqrt{(n1r-r^2)/m}$, given in [12] is plotted, too. We observe that in our experiments IPLR-GS_P recovers the sought matrix with RMSE values always smaller than $1.3RMSE_{or}$, despite the condition number of the matrix. This is not the case for OptSpace and Incremental OptSpace; OptSpace can reach a comparable accuracy only if the percentage of known entries exceeds $30\%$. As expected, for all methods the error decreases as the number of subsampled entries increases.

In summary, for mildly ill-conditioned random matrices our approach is more reliable than OptSpace and Incremental OptSpace as the latter algorithms might struggle with computing the singular vectors of the sparsified data matrix accurately, and they cannot deliver precision comparable to that of IPLR. For the sake of completeness, we remark that we have tested OptSpace also on the well-conditioned random matrices reported in Tables 2, 4 and 3, 5. On these problems IPLR and OptSpace provide comparable solutions, but as a solver specially designed for matrix-completion problems OptSpace is generally faster than IPLR.

### Rank Updating

We now test the effectiveness of the rank updating/downdating strategy described in Algorithm 2. To this purpose, we run IPLR-GS_P starting from $r=1$, with rank increment/decrement $\delta _r = 1$ and report the results in Table 6 for ${\hat{n}}=600,800,1000$. In all runs, the target rank has been correctly identified by the updating strategy and the matrix *B* is well-recovered. Runs in italic have been obtained allowing 10 inner Gauss–Seidel iterations. In fact, 5 inner Gauss–Seidel iterations were not enough to sufficiently reduce the residual in (2.4) and the procedure did not terminate with the correct rank. Comparing the values of the cpu time in Tables 2 and 6 we observe that the use of rank updating strategy increases the overall time; on the other hand, it allows to adaptively modify the rank in case a solution of (5.4) with the currently attempted rank does not exist.

**Table 6:** IPLR-GS_P on random matrices starting with $r=1$

rank/*n*/*m*	IPLR-GS_P				
	$\Vert {{\mathcal {A}}}(X)-b\Vert$	$\Vert XS-\mu I\Vert _F$	$\lambda _{\min }(S)$	${{\mathcal {E}}}$	cpu
3/1200/35910	4E−04	1E−03	4E−08	3E−06	161
4/1200/47840	4E−04	1E−03	5E−08	3E−06	206
5/1200/59750	5E−05	1E−03	5E−08	3E−07	315
6/1200/71640	9E−06	1E−03	4E−08	5E−08	390
7/1200/83510	8E−06	1E−03	4E−08	4E−08	494
8/1200/95360	*4E*−*07*	*1E*−*03*	*4E*−*08*	*2E*−*09*	*746*
3/1600/57492	4E−04	1E−03	3E−08	3E−06	411
4/1600/76608	3E−04	1E−03	4E−08	1E−06	488
5/1600/95700	7E−05	1E−03	3E−08	3E−07	641
6/1600/114768	2E−05	1E−03	3E−08	8E−08	841
7/1600/133812	*4E*−*07*	*1E*−*03*	*3E*−*08*	*1E*−*09*	*996*
8/1600/152832	*1E*−*07*	*1E*−*03*	*3E*−*08*	*4E*−*10*	1238
3/2000/83874	3E−04	1E−03	3E−08	2E−06	566
4/2000/111776	3E−04	1E−03	3E−08	1E−06	791
5/2000/139650	3E−05	1E−03	3E−08	1E−07	894
6/2000/167496	9E−06	1E−03	3E−08	1E−08	1293
7/2000/195314	3E−07	1E−03	3E−08	1E−7	1809
8/2000/223104	*1E*−*07*	*1E*−*03*	*3E*−*08*	*3E*−*10*	*2149*

**Fig. 8:**
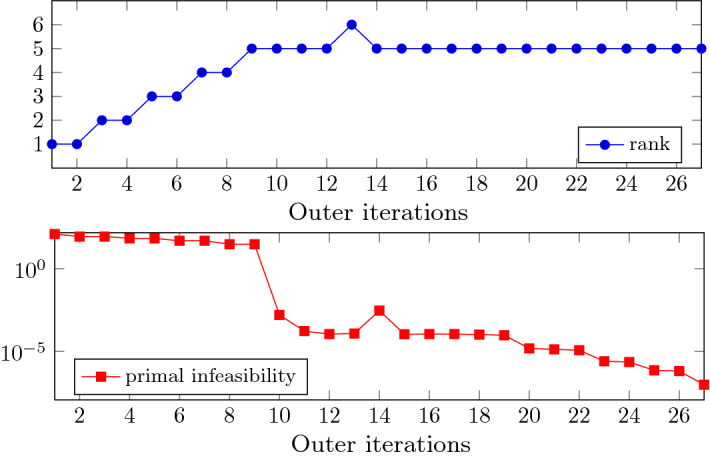
Typical behaviour of the rank update strategy described in Algorithm 2. Data: ${\hat{n}}=50$, target rank $r=5$, starting rank $r=1$

The typical updating behaviour is illustrated in Fig. 8 where we started with rank 1 and reached the target rank 5. In the first eight iterations a solution of the current rank does not exist and therefore the procedure does not manage to reduce the primal infeasibility as expected. Then, the rank is increased. At iteration 9 the correct rank has been detected and the primal infeasibility drops down. Interestingly, the method attempted rank 6 at iteration 13, but quickly corrected itself and returned to rank 5 which was the right one.

The proposed approach handles well the situation where the matrix which has to be rebuilt is nearly low-rank. We recall that by Corollary 5.2 we generate a low-rank approximation ${\bar{X}}_k$, while the primal variable $X_k$ is nearly low-rank and gradually approaches a low-rank solution. Then, at termination, we approximate the nearly low-rank matrix that has to be recovered with the low-rank solution approximation.

Letting $\sigma _1\ge \sigma _2\ge \dots \ge \sigma _{{\hat{n}}}$ be the singular values of *B*, we perturbed each singular value of *B* by a random scalar $\xi = 10^{-3}\eta$, where $\eta$ is drawn from the standard normal distribution, and using the SVD decomposition of *B* we obtain a nearly low-rank matrix ${\hat{B}}$. We applied IPLR-GS_P to (5.4) with the aim to recover the nearly low-rank matrix ${\hat{B}}$ with tolerance in the stopping criterion set to $\epsilon =10^{-4}$. Results reported in Table 7 are obtained starting from $r=1$ in the rank updating strategy. In the table we also report the rank ${\bar{r}}$ of the rebuilt matrix ${\bar{X}}$. The run corresponding to rank 8, in italic in the table, has been performed allowing a maximum of 10 inner Gauss–Seidel iterations. We observe that the method always rebuilt the matrix with accuracy consistent with the stopping tolerance. The primal infeasibility is larger than the stopping tolerance, as data *b* are obtained sampling a matrix which is not low-rank and therefore the method does not manage to push primal infeasibility below $10^{-3}$. Finally we note that in some runs (rank equal to 4,5,6) the returned matrix ${\bar{X}}$ has a rank ${\bar{r}}$ larger than that of the original matrix *B*. However, in this situation we can observe that ${\bar{X}}$ is nearly-low rank as $\sigma _i=O(10^{-3})$, $i=r+1,\ldots ,{\bar{r}}$ while $\sigma _i \gg 10^{-3}$, $i=1,\ldots ,r$. Therefore the matrices are well rebuilt for each considered rank *r* and the presence of small singular values does not affect the updating/downdating procedure.

**Table 7:** IPLR-GS_P starting from $r=1$ on nearly low-rank matrices ($\xi = 10^{-3}$)

rank/*n*/*m*	IPLR-GS_P					
	$\Vert {{\mathcal {A}}}(X)-b\Vert$	$\Vert XS-\mu I\Vert _F$	$\lambda _{\min }(S)$	$\Vert {\bar{X}} - {\hat{B}}\Vert _F /\Vert {\hat{B}}\Vert _F$	${\hat{r}}$	cpu
3/1200/35910	4E−03	1E−03	4E−08	2E−05	3	218
4/1200/47840	5E−03	1E−03	4E−08	2E−05	**5**	506
5/1200/59750	5E−03	2E−03	1E−07	2E−05	**7**	937
6/1200/71640	6E−03	1E−03	4E−08	2E−05	**7**	797
7/1200/83510	6E−03	1E−03	4E−08	2E−05	7	642
8/1200/95360	*7E*−*03*	*1E*−*03*	*4E*−*08*	*2E*−*05*	8	1173

### Tests on Real Data Sets

In this section we discuss matrix completion problems arising in diverse applications as the matrix to be recovered represents city-to-city distances, a grayscale image, game parameters in a basketball tournament and total number of COVID-19 infections.

### Low-Rank Approximation of Partially Known Matrices

We now consider an application of matrix completion where one wants to find a low-rank approximation of a matrix that is only partially known.

As the first test example, we consider a $312 \times 312$ matrix taken from the “City Distance Dataset” [10] and used in [11], that represents the city-to-city distances between 312 cities in the US and Canada computed from latitude/longitude data.

We sampled the 30% of the matrix *G* of geodesic distances and computed a low-rank approximation ${\bar{X}}$ by IPLR-GS_P inhibiting rank updating/downdating and using $\epsilon =10^{-4}$. We compared the obtained solution with the approximation ${\bar{X}}_{os}$ computed by OptSpace and the best rank-*r* approximation ${\bar{X}}_r$, computed by truncated SVD (TSVD), that requires the knowledge of the full matrix *G*. We considered some small values of the rank ($r=3,4,5$) and in Table 8 reported the errors ${{\mathcal {E}}}_{ip}=\Vert G- {\bar{X}}\Vert _F/\Vert G\Vert _F$, ${{\mathcal {E}}}_{os}=\Vert G-{\bar{X}}_{os}\Vert _F/\Vert G\Vert _F$ and ${{\mathcal {E}}}_r=\Vert G-{\bar{X}}_r\Vert _F/\Vert G\Vert _F$. We remark that the matrix *G* is not nearly-low-rank, and our method correctly detects that there does not exist a feasible rank *r* matrix as it is not able to decrease the primal infeasibility below 1*e*0. On the other hand the error ${{\mathcal {E}}}_{ip}$ in the provided approximation, obtained using only the 23% of the entries, is the same as that of the best rank-*r* approximation ${\bar{X}}_r$. Note that computing the 5-rank approximation is more demanding. In fact the method requires on average: 3.4 Gauss–Seidel iterations, 37 unpreconditioned CG iterations for computing $\Delta U$ and 18 preconditioned CG iterations for computing $\Delta y$. In contrast, the 3-rank approximation requires on average: 3.8 Gauss–Seidel iterations, 18 unpreconditioned CG iterations for computing $\Delta U$ and 10 preconditioned CG iterations for computing $\Delta y$. As a final comment, we observe that IPLR-GS fails when $r=5$ since unpreconditioned CG struggles with the solution of (4.14). The computed direction $\Delta y$ is not accurate enough and the method fails to maintain *S* positive definite within the maximum number of allowed backtracks. Applying the preconditioner cures the problem because more accurate directions become available. Values of the error ${{\mathcal {E}}}_{op}$ obtained with OptSpace are larger than ${{\mathcal {E}}}_r$. However it is possible to attain comparable values for $r=3$ and $r=5$ under the condition that the default maximum number of iterations of OptSpace is increased 10 times. In these cases, OptSpace is twice and seven time faster, respectively.

**Table 8:** TSVD, OptSpace and IPLR-GS_P for low rank approximation of the City Distance matrix

Rank	TSVD	OptSpace	IPLR-GS_P				
	${{\mathcal {E}}}_r$	${{\mathcal {E}}}_{op}$	${{\mathcal {E}}}_{ip}$	$\Vert {{\mathcal {A}}}(X)-b\Vert$	$\Vert XS-\mu I\Vert _F$	$\lambda _{\min }(S)$	cpu
3	1.15E−01	1.97E−01	1.23E−01	4E00	8E−04	4E−07	48
4	7.06E−02	1.99E−01	7.85E−02	3E00	8E−04	4E−07	70
5	5.45E−02	1.30E−01	6.01E−02	2E00	8E−04	4E−07	243

**Fig. 9:**
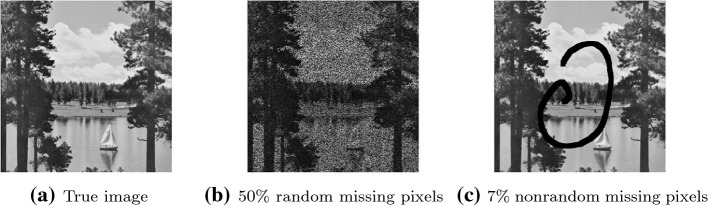
The Lake test true image and the inpainted versions

As the second test example, we consider the problem of computing a low rank approximation of an image that is only partially known because some pixels are missing and we analyzed the cases when the missing pixels are distributed both randomly and not randomly (inpainting). To this purpose, we examined the *Lake* $512\times 512$ original grayscale image^2^ shown in Fig. 9c and generated the inpainted versions with the 50% of random missing pixels (Fig. 9b) and with the predetermined missing pixels (Fig. 9c).

We performed tests fixing the rank to values ranging from 10 to 150 and therefore used IPLR-BB which is computationally less sensitive than IPLR-GS to the magnitude of the rank.

**Fig. 10:**
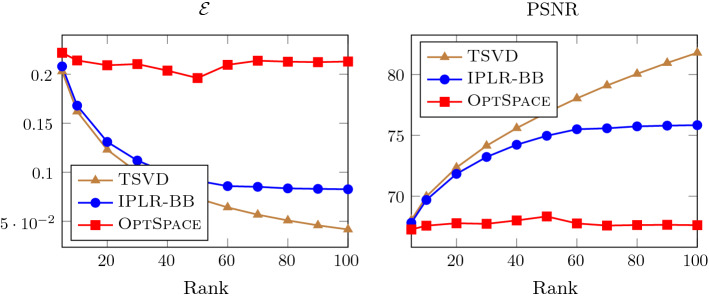
Rank versus error and PSNR of the Lake image recovered with truncated SVD (TSVD), IPLR-BB and OptSpace (50% random missing pixels in Fig. 9b)

In Fig. 10 we plot the quality of the reconstruction in terms of relative error ${{\mathcal {E}}}$ and PSNR (Peak-Signal-to-Noise-Ratio) against the rank, for IPLR-BB, OptSpace and truncated SVD. We observe that when the rank is lower than 40, IPLR-BB and TSVD give comparable results, but when the rank increases the quality obtained with IPLR-BB does not improve. As expected, by adding error information available only from the knowledge of the full matrix, the truncated SVD continues to improve the accuracy as the rank increases. The reconstructions produced with OptSpace display noticeably worse values of the two relative errors (that is, larger ${{\mathcal {E}}}$ and smaller PSNR, respectively) despite the rank increase.

**Fig. 11:**
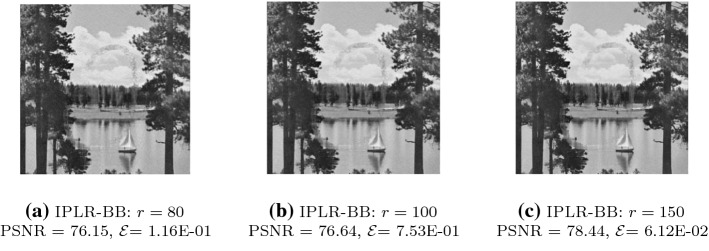
Images recovered by IPLR-BB for different rank values and corresponding PSNR and error (non-random missing pixels in Fig. 9c)

Figure 11 shows that IPLR-BB is able to recover the inpainted image in Fig. 9c and that visually the quality of the reconstruction benefits from a larger rank. Images restored by OptSpace are not reported since the relative PSNR values are approximately 10 points lower than those obtained with IPLR-BB. The quality of the reconstruction of images Fig. 9b and c obtained with OptSpace cannot be improved even if the maximum number of iterations is increased tenfold.

### Application to Sports Game Results Predictions

**Fig. 12:**
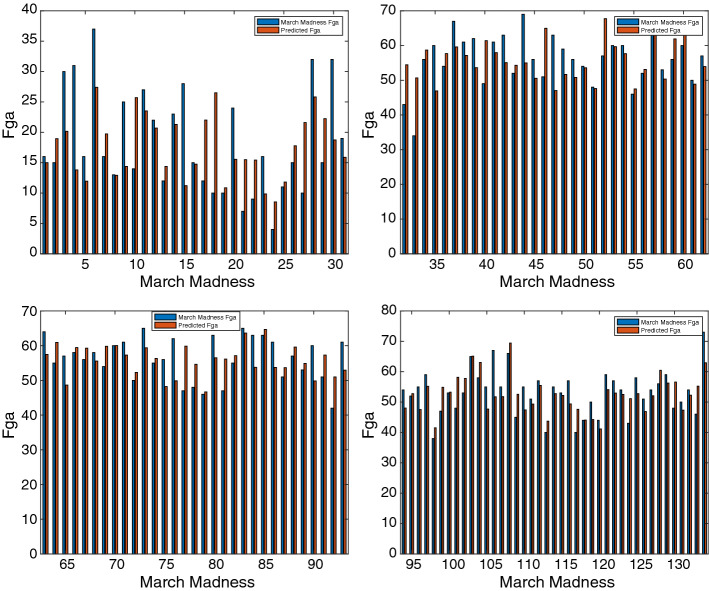
Predicted and March Madness FGA statistics. Top-Left: matches 1 to 31, Top-Right matches 32 to 62, Bottom-Left matches 63 to 93, Bottom-Right matches 94 to 134

Matrix completion is used in sport predictive models to forecast match statistics [27]. We consider the dataset concerning the NCAA Men’s Division I Basketball Championship, in which each year 364 teams participate.^3^ The championship is organized in 32 groups, called Conferences, whose winning teams face each other in a final single elimination tournament, called March Madness. Knowing match statistics of games played in the regular Championship, the aim is to forecast the potential statistics of the missing matches played in the March Madness phase. In our tests, we have selected one match statistic of the 2015 Championship, namely the fields goals attempted (FGA) and have built a matrix where teams are placed on rows and columns and nonzero *ij*-values correspond to the FGA made by team *i* and against team *j*. In this season, only 3771 matches were held and therefore we obtained a rather sparse $364\times 364$ matrix of FGA statistics; in fact, only the 5.7% of entries of the matrix that has to be predicted is known. To validate the quality of our predictions we used the statistics of the 134 matches actually played by the teams in March Madness. We verified that in order to obtain reasonable predictions of the missing statistics the rank of the recovered matrix has to be sufficiently large. Therefore we use IPLR-BB setting the starting rank $r=20$, rank increment $\delta _r=10$ and $\epsilon =10^{-3}$. The algorithm terminated recovering matrix ${\bar{X}}$ of rank 30. In Fig. 12 we report the bar plot of the exact and predicted values for each March Madness match. The matches have been numbered from 1 to 134. We note that except for 12 mispredicted statistics, the number of fields goals attempted is predicted reasonably well. In fact, we notice that the relative error between the true and the predicted statistic is smaller than $20\%$ in the 90% of predictions.

On this data set, OptSpace gave similar results to those in Fig. 12 returning a matrix of rank 2.

### Application to COVID-19 Infections Missing Data Recovery

**Fig. 13:**
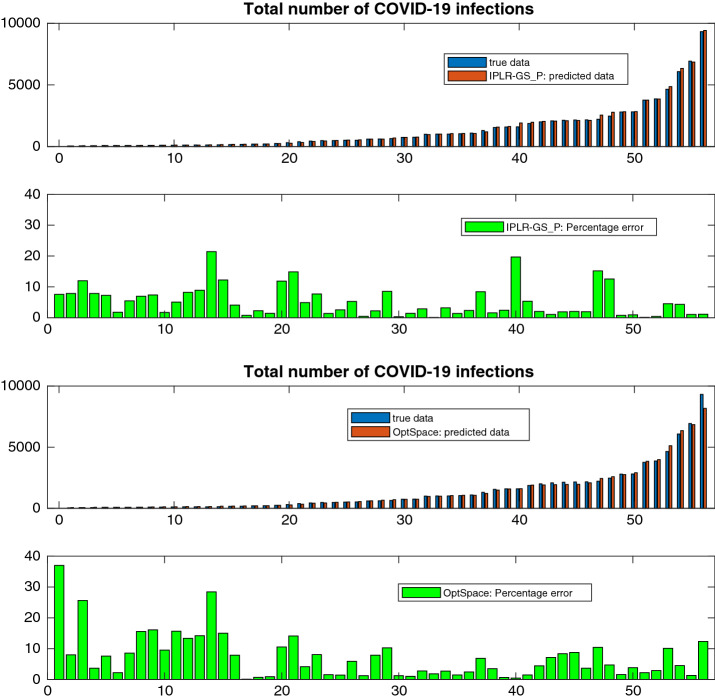
Predicted and actual number of COVID-19 infections (top) and corresponding percentage error , obtained with IPLR-GS_P (2 top plots) and OptSpace (2 bottom plots)

We now describe a matrix completion problem where data are the number of COVID-19 infections in provincial capitals of regions in the North of Italy. Each row and column of the matrix corresponds to a city and to a day, respectively, so that the *ij*-value corresponds to the total number of infected people in the city *i* on the day *j*. We used data made available by the Italian Protezione Civile^4^ regarding the period between March 11th and April 4th 2020, that is, after restrictive measures have been imposed by the Italian Government until the date of paper submission. We assume that a small percentage (5%) of data is not available to simulate the real case because occasionally certain laboratories do not communicate data to the central board. In such a case our aim is to recover this missing data and provide an estimate of the complete set of data to be used to make analysis and forecasts of the COVID-19 spread. Overall, we build a $47 \times 24$ dense matrix and attempt to recover 56 missing entries in it. We use IPLR-GS_P with starting rank $r=2$, rank increment $\delta _r=1$ and $\epsilon =10^{-4}$ and we have obtained a matrix ${\bar{X}}$ of rank 2. The same rank is obtained using OptSpace but only if the maximum number of its iterations is increased threefold. In Fig. 13 both the predicted and actual data (top) and the percentage error (bottom) are plotted using the two solvers. We observe that IPLR-GS_P yields an error below 10% except for 8 cases and in the worst case it reaches 22%. The error obtained with OptSpace exceeds 10% in 15 cases and in one case reaches 37%.

The good results obtained with IPLR-GS_P for this small example are encouraging for applying the matrix completion approach to larger scale data sets.

## Conclusions

We have presented a new framework for an interior point method for low-rank semidefinite programming. The method relaxes the rigid IPM structure and replaces the general matrix *X* with the special form (2.3) which by construction enforces a convergence to a low rank solution as $\mu$ goes to zero. Therefore effectively instead of requiring a general $n \times n$ object, the proposed method works with an $n \times r$ matrix *U*, which delivers significant storage and cpu time savings. It also handles well problems with noisy data and allows to adaptively correct the (unknown) rank. We performed extensive numerical tests on SDP reformulation of matrix completion problems using both the first- and the second-order methods to compute search directions. The convergence of the method has been analysed under the assumption that eventually the steplength $\alpha _k$ is equal to one (Assumption 1). However, this seemingly strong assumption does hold in all our numerical tests except for the sports game results predictions where the number of known entries of the matrix is extremely low.

Our numerical experience shows the efficiency of the proposed method and its ability to handle large scale matrix completion problems and medium scale problems arising in real-life applications. A comparison with OptSpace reveals that the proposed method is versatile and it delivers more accurate solutions when applied to ill-conditioned or to some classes of real-life applications. It is generally slower than methods specially designed for matrix completion as OptSpace, but our method has potentially a wider applicability.

## Appendix A. Notes on Kronecker Product and Matrix Calculus

Let us also recall several useful formulae which involve Kronecker products. For each of them, we assume that matrix dimensions are consistent with the multiplications involved.

Let *A*, *B*, *C*, *D* be matrices of suitable dimensions. Then

A.1 $$\begin{aligned} (A \otimes B) (C\otimes D)= & {} (AC \otimes BD) \end{aligned}$$

A.2 $$\begin{aligned} vec(AXB)= & {} (B^T \otimes A) vec(X) \end{aligned}$$

A.3 $$\begin{aligned} vec(AX^TB)= & {} (B^T \otimes A) vec(X^T) = (B^T \otimes A) \Pi vec(X) , \end{aligned}$$

where $\Pi$ is a permutation matrix which transforms *vec*(*X*) to $vec(X^T)$. Moreover, assume that *A* and *B* are square matrices of size *n* and *m* respectively. Let $\lambda _1, \dots , \lambda _n$ be the eigenvalues of *A* and $\mu _1, \dots , \mu _m$ be those of *B* (listed according to multiplicity). Then the eigenvalues of $A \otimes B$ are

$$\begin{aligned} \lambda _{i}\mu _{j},\qquad i=1,\ldots ,n,\,j=1,\ldots ,m. \end{aligned}$$

Finally, following [18], we recall some rules for derivatives of matrices that can be easily derived applying the standard derivation rules for vector functions (chain rule, composite functions) and identifying $d\, \mathcal {G}(X)/d\,(X)$ by using the vectorization $d \,vec \mathcal {G}(X)/ d\, vec(X)$, where $\mathcal {G}(X)$ is a matrix function. In particular we have that given the matrices $A \in \mathbb {R}^{n\times m}$, $B \in \mathbb {R}^{p\times q}$ and *X* defined accordingly, it holds

$$\begin{aligned} \frac{d\, AA^T}{d\, A}= & {} (A \otimes I_n) + (I_n\otimes A),\\\frac{d\, AXB}{d\,X}= & {} (B^T \otimes A). \end{aligned}$$

## Footnotes

1 OptSpace: http://swoh.web.engr.illinois.edu/software/optspace/code.html.

2 The Lake image can be downloaded from http://www.imageprocessingplace.com.

3 The March Machine Learning Mania dataset is available in the website https://www.kaggle.com/c/march-machine-learning-mania-2016/data.

4 The dataset is available at https://github.com/pcm-dpc/COVID-19/tree/master/dati-province.
